# PAM-OBG: A monoamine oxidase B specific prodrug that inhibits MGMT and generates DNA interstrand crosslinks, potentiating temozolomide and chemoradiation therapy in intracranial glioblastoma

**DOI:** 10.18632/oncotarget.25246

**Published:** 2018-05-08

**Authors:** Martyn A. Sharpe, Sudhir Raghavan, David S. Baskin

**Affiliations:** ^1^ Department of Neurosurgery, Kenneth R. Peak Brain and Pituitary Tumor Center, Houston Methodist Hospital, TX 77030, Houston, USA

**Keywords:** glioblastoma, MGMT, MAOB, drug, chemotherapy

## Abstract

Via extensive analyses of genetic databases, we have characterized the DNA-repair capacity of glioblastoma with respect to patient survival. In addition to elevation of O^6^-methylguanine DNA methyltransferase (MGMT), down-regulation of three DNA repair pathways; canonical mismatch repair (MMR), Non-Homologous End-Joining (NHEJ), and Homologous Recombination (HR) are correlated with poor patient outcome.

We have designed and tested both *in vitro* and *in vivo*, a monoamine oxidase B (MAOB) specific prodrug, PAM-OBG, that is converted by glioma MAOB into the MGMT inhibitor O^6^-benzylguanine (O^6^BG) and the DNA crosslinking agent acrolein. In cultured glioma cells, we show that PAM-OBG is converted to O^6^BG, inhibiting MGMT and sensitizing cells to DNA alkylating agents such as BCNU, CCNU, and Temozolomide (TMZ). In addition, we demonstrate that the acrolein generated is highly toxic in glioma treated with an inhibitor of Nucleotide Excision Repair (NER).

In mouse intracranial models of primary human glioma, we show that PAM-OBG increases survival of mice treated with either BCNU or CCNU by a factor of six and that in a chemoradiation model utilizing six rounds of TMZ/2Gy radiation, pre-treatment with PAM-OBG more than doubled survival time.

## INTRODUCTION

### GBM

Gliomas are the most common primary tumors that arise within the brain and have histologic features similar to normal glial cells (i.e., astrocytes, oligodendrocytes, and ependymal cells). The incidence rate of primary malignant brain tumors is about 6.5 cases per 100,000/year (2005–2009) [1]. In the USA the incidence of glioblastoma multiforme (GBM), grade IV glioma, is about 3.19 per 100,000 per year. The outcome for GBM patients is grim, with a median survival of 16.7 months and only 30% of patients surviving ≥ 24 months [2].

### Current solutions are inadequate

#### Initial therapy

A graphic indicating the therapy and temporal pattern of administration in typical primary and salvage therapies of GBM is to be found in Supplementary Figure 1.

The Stupp protocol is the standard of care for the treatment of GBM and has led to significant survival improvements since its publication in 2005 [4, 5]. The original Stupp protocol consists of an aggressive resection of the GBM. Patients then undergo chemoradiotherapy some 2–8 weeks later; a combination of focal radiation (tumor volume + 2–3 cm margins) in 2 Gy daily fractions (5 days per week, typically Monday to Friday) for 6 weeks and TMZ, 75 mg/m^2^/day 1–2 hours before the radiation, for six weeks, with TMZ also taken during weekends. TMZ is then given post-radiotherapy (adjuvant) in six cycles consisting of 150–200 mg/m^2^/day for 5 days during each 28-day cycle; Supplementary Figure 1A. The main modification to this standard of care is the use of chemotherapy with radiotherapy, rather than after radiotherapy. The Stupp regime, in those patients that could tolerate the high radiation and TMZ, resulted in a significant survival improvement with 2-year-survival increasing from 10.4 to 26.5%.

Use of alkylating agents in GBM salvage therapy: Gliomas eventually develop resistance toward TMZ and patients then undergo salvage therapy that typically involves either additional chemotherapy or a combination of chemotherapy and re-radiation therapy. The alkylating agent CCNU is still a popular choice for salvage therapy, given as a single agent or part of a drug cocktail, with or without radiation. Two recent trials by Batchelor [3]. and by Taal [4]. use high dosages of CCNU, 56-days apart, to treat TMZ-resistant GBM, Supplementary Figure 1B. Dosage staggering is required because all alkylating agents attack bone marrow and therefore life-threatening complications are common. CCNU extends survival by ≈ 9.5 months, in patients who can tolerate the regime. PCV therapy is an extensively used salvage therapy and consists of Procarbazine (an alkylating agent), CCNU and Vincristine (tubulin disruptor), with drugs given in a specific sequence, over a 56-day cycle, Supplementary Figure 1C. Outcomes are slightly worse than aggressive CCNU therapy, with survival extended by ≈ 8.5 months [5–7], but this therapy is better tolerated by patients. Van den Bent and co-workers demonstrated that staggered doses of BCNU extended survival by 9.3 months in GBM patients with acquired TMZ resistance [8], but using the same protocol with a larger patient cohort extended survival by only 5.5 months [9], Supplementary Figure 1D. A trial by Hayat [10] of patients with either low-grade glioma (LGG) or GBM tumors used concomitant CCNU and re-radiation, followed by adjuvant CCNU. The low doses of CCNU caused few off-target complications and coupled with focal radiation of the tumor extended survival by ≈ 8 months, Supplementary Figure 1E.

### The basis of chemotherapy: how alkylating agents work as chemotherapeutic drugs; alkylation and oxidative stress

Chemotherapeutic alkylating agents work because they alkylate a wide range of cellular nucleophiles such as thiols, alcohols and amines, including the nucleotide bases which make up DNA. Resistance to alkylating agents is typically due to one or more DNA repair systems being upregulated (repair function), or a pro-apoptotic signaling pathway (report function) linked to DNA repair being down-regulated in cancer cells [11, 12]. This means that DNA damage induced by a chemotherapeutic alkylating agents is either being repaired or tolerated in cancer cells more than in some of the non-cancerous cells, typically bone marrow, which is normally the most sensitive off-target tissue.

Elevated rates of reactive oxygen species (ROS) production are a hallmark of most cancer cells where they are associated with tumor development and progression [13]. Elevated steady-state ROS levels cause cancer cells to upregulate many DNA repair pathway proteins (reviews [14–16] and ROS preconditioning in glioma may be a driving force in innate drug-resistant phenotypes [17].

### Why mono-therapeutic agents fail against glioma: evolution

Cancer cells, especially those present in a high-grade tumor, are the end products of evolutionary selection pressure that has acted on the descendants which arose from a single deviant cell. One can envision cancers as asexual pathogens. Computation evolutionary simulations and studies of asexual eukaryotic pathogens indicate that high mutation rates and low fidelity in DNA repair are hallmarks of asexual pathogens that have ‘evolved to evolve’ [18, 19]. High-grade tumors are heterogeneous, an indicator of rapid evolution, and tumor cells are characterized by high mutation loads, amplifications, deletions and structural rearrangements of swathes of DNA [20–24]. We postulated that aberrations in DNA repair pathways would be selected for in GBM, allowing these cancer cells to undergo rapid evolution and niche exploitation, but could be its ‘Achilles heel’ for a highly targeted chemotherapeutic drug strategy.

As an aid to the discussion of DNA repair pathways we have prepared graphics illustrating the mechanistic aspect of the Base Excision Repair (BER), Mismatch Repair (MMR) and Nucleotide Excision Repair (NER), which are shown in Supplementary Figure 2. As the Non-Homologous End-Joining (NHEJ), Micro-homology-Mediated End Joining (MMEJ) and Homologous Recombination (HR) and nucleophosmin-linked translesion DNA synthesis pathway in GBM [25] do not merit highly in the context of PAM-OBG treatment and we will not cover them in detail. We recommend the website maintained by KEGG.org for further information [26].

### TMZ resistance mechanisms in glioma

**Figure 1:**
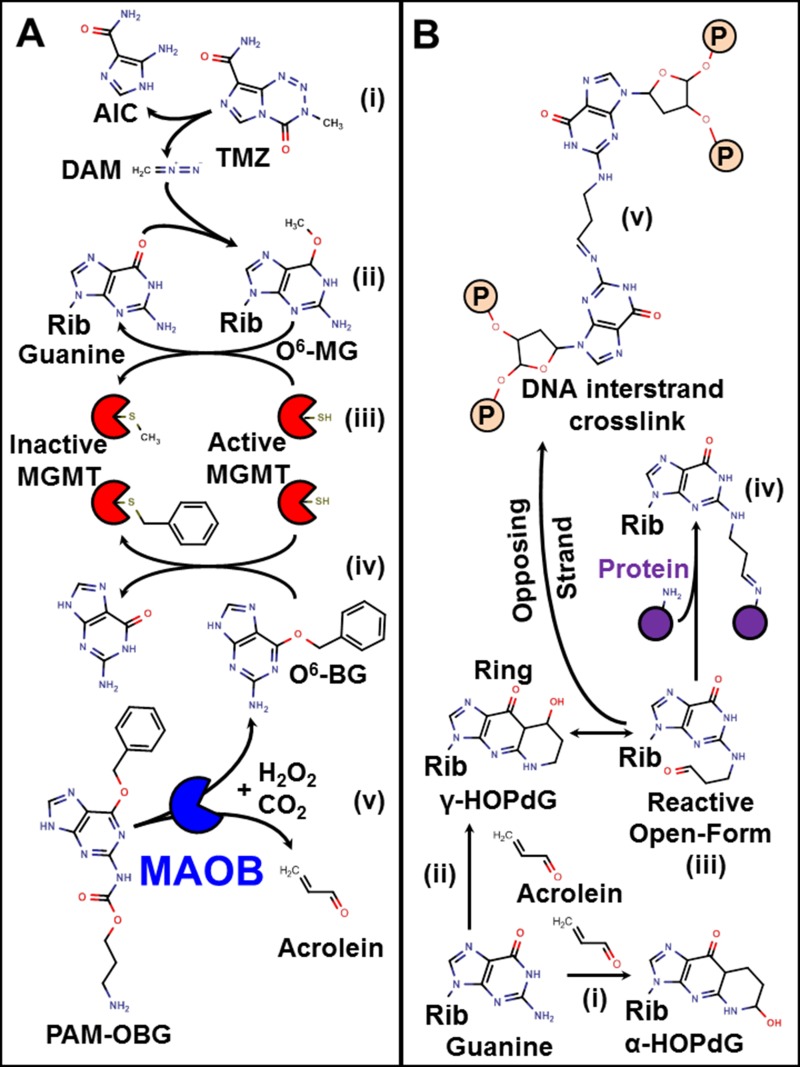
Mechanism of action of TMZ, O^6^-BG, PAM-OBG, and acrolein (**A**) shows the action of TMZ, O^6^BG, and PAM-OBG. TMZ is a prodrug that spontaneously gives rise to the methylation agent diazomethane (i). DAM can react with DNA giving rise to O^6^MeG (ii). This toxic lesion is removed by MGMT which transfers the methyl adduct to a thiol in its active site (iii). MGMT can also react with O^6^BG, being deactivated, with a benzyl thio-ether formed (iv). PAM-OBG is oxidized by MAOB, producing CO2, O^6^BG, and acrolein (v). The reactions of acrolein and DNA are shown in (**B**). Guanine residues react with acrolein to generate HOPdG, the α- HOPdG bulky adduct is stable (i), but the γ-HOPdG has unreactive closed (ii) and open (iii) forms. Proteins, including DNA repair proteins, can become covalently linked to γ-HOPdG (iv), but the formation of DNA interstrand crosslinks (v) is the more toxic lesion.

TMZ is a prodrug version of the highly reactive methylation agent diazomethane, (Figure 1A (i)) which gives rise to a large number of DNA base adducts, including O^6^-methylguanine (O^6^MeG), Figure 1A (ii).

In GBM the action of the enzyme O^6^-methylguanine DNA methyltransferase (MGMT) is the primary means of TMZ resistance. MGMT is a small suicide-enzyme that directly removes alkyl adducts from O^6^-guanine, via direct transfer of the alkyl to a cysteine thiol in the enzymes active site, Figure 1A (iii). Each MGMT can only repair a single lesion, as after adduct transfer and formation of alkyl thioether, MGMT is inactivated. MGMT also accepts an alkyl group from specific inhibitors such as O^6^-benzylguanine (O^6^BG), Figure 1A (iv).

MGMT repairs the O^6^MeG lesions generated by TMZ, *in vitro* and *in vivo*, limiting TMZ toxicity. MGMT gene expression levels are highly dependent on MGMT promoter methylation status, and in primary GBM biopsy samples, promoter methylation is found in some 35–45% of samples, with methylation of the promoter indicative of low MGMT mRNA levels [27–29]. However, mRNA levels do not directly correlate with the steady-state levels of the active MGMT, and this may in part be due to post-translational modification, including phosphorylation [30], altering MGMT biological half-life.

### Levels of active MGMT in GBM

The steady-state levels of active MGMT in treatment naïve tumors depend on the relative rate of synthesis and rate of degradation, which is governed by ubiquitination rate. STAT3 activation causes phosphorylation of MGMT and this enhances MGMT lifetime, raising the steady-state levels of active MGMT some 5-fold [31]. MGMT/pMGMT levels correlate with TMZ treatment failure in GBM [31–41]. Thus, activated STAT3 may be a major mechanism of MGMT induced resistance to alkylating agents [31, 32].

Correlation of MGMT protein levels and MGMT promoter methylation is not statistically significant, and there is also no significant survival difference between patients whose GBM biopsy samples had high or low levels of MGMT if the MGMT is determined immunohistochemically [33, 34].

### Mechanisms TMZ resistance

Evaluations of the changes in DNA-repair mechanisms in GBM tumors resected prior to chemoradiation, and then following failure of the standard Stupp protocol, indicates that upregulation of MGMT is not always the basis of TMZ drug resistance.

Generally, it appears that resistance to TMZ appears to arise from three mechanisms:

1) Upregulation of MGMT [28, 34–37].

2) Downregulation of canonical MMR (MutSa/MutLa). Agarwal [38] noted that after TMZ therapeutic failure there was generally an upregulation of MGMT or there was a downregulation of canonical MMR (or mutations in MutSa/MutLa) in second resection tumor samples.

3) Upregulation of BER, specifically the action of N-Methylpurine DNA Glycosylase (MPG), and its interaction with the Ataxia Telangiectasia Mutated Serine/Threonine Kinase (ATM) signaling system [39, 40]. Agnihotri and co-workers demonstrated, using samples drawn from pediatric GBM patients, that ATM/MPG form a complex and that MPG-BER may be able to remove O^6^MeG bases when MPG is phosphorylated via ATM [40].

### Failure of MGMT inhibitors in the clinical setting

O^6^BG, a potent inhibitor of MGMT, is nontoxic when administered to animals or humans as a single agent [41]. A phase III trial utilizing O^6^BG to ablate MGMT activity throughout a patient’s tissues, prior to radiotherapy/BCNU + BCNU, was highly disappointing as O^6^BG did not provide an added benefit, but instead caused additional toxicity. Hematologic Grade 4 or higher toxicities quadrupled in patients receiving O^6^BG & BCNU vs. the BCNU arm. Several phase II trials utilizing an MGMT inhibitor combined with radiotherapy/TMZ have failed [42–44].

The combination of alkylating agents with an MGMT inhibitor has failed in clinical trials for a simple reason; alkylating agents are more toxic to non-target tissues, especially bone marrow, than toward tumor cells.

### A MAOB activated bifunctional prodrug targeting MGMT and DNA

#### Design philosophy

Our aim was to design a therapeutic agent that would complement the existing best clinical treatment regime, potentiating TMZ chemoradiation toxicity within glioma cells but not systemically, making a chemotherapeutic to fit directly into the Stupp regime.

### Strategy for a MAOB activated MGMT inhibitor

Expression of MAOB in LGG and GBM is highly elevated, and its levels correlate with the degree of tumor hypoxia [45–47]. Data from the TCGC database indicates that all major glioma sub-types have a similar MAOB mRNA distribution, with the minimum showing > 2.5-fold the level of normal brain tissue, the average ≈ 8-fold greater, and maximal > 30-fold higher (166 GBM, 283 LGG-WT, and 233 LGG-IDH mut) [48, 49]. Additionally, we find that MAOB and MGMT expression levels are highly correlated in GBM (*p* < 0.01) [48, 49]. We envisioned a prodrug that would become active after oxidation by MAOB and that after maturation would generate O^6^BG to inhibit MGMT would sensitize cells to TMZ chemoradiation. As the oxidative activation of a prodrug by MAOB also generates an aldehyde, we sought a design whereby the aldehyde produced from the prodrug maturation would be a DNA damaging agent in its own right.

### Choice of MAOB as a maturation enzyme

Human bone marrow has almost no expression of MAOB, which makes a MAOB-based prodrug designed to potentiate alkylating agents highly attractive. Human MAOB and MGMT mRNA expression level [50], activity [51] and also protein half-life [52–54] are highly tissue dependent. Supplementary Figure 3A shows the levels of MGMT and MAOB mRNA (normalized to the liver) measured in human tissues [50, 55]. The MGMT:MAOB ratio of a tissue is a proxy for the off-target sensitivity expected from a MAOB-activated MGMT inhibitor, Supplementary Figure 3B. The very high levels of MGMT and low levels MAOB in bone marrow suggest that this would be the body’s least sensitive tissue.

### MAOB maturation chemistry

We have identified two major MAO-specific prodrug chemical motifs that can be used in MAO specific prodrug design; tetrahydropyridines that become mitochondrial ‘smart bombs’ [56, 57] and immolative amine linkers that undergo ‘unmasking’. We have used the latter for the design of PAM-OBG, which allows an inactive ‘masked’ adduct of O^6^BG to be converted in O^6^BG only inside cells with MAOB activity. MAO’s oxidize propylamine ethers to give rise to acrolein and a primary alcohol, and this mechanism is the basis of the MAO-Glo™ (Luciferin) Assay (Promega Corporation, Madison, WI) [58, 59].

PAM-OBG is a MAOB specific prodrug version of O^6^BG that uses a similar immolative ‘unmasking’ route as used in MAO-Glo™. In PAM-OBG an amide-ether (carbamate) to a primary amine is used as the immolative propylamine linker, rather than using an ether linkage to a primary alcohol, as in the case of the aminopropylether luciferin MAO substrate used in MAO-Glo™.

Figure 1A (v) shows that PAM-OBG consists of an MAO-oxidizable propylamine linked to the 2-amino position of O^6^BG, via a carbamate. *In silico* modeling using the O^6^MeG hMGMT (C145S) crystal structure [60]. indicated that the addition to the 2-amino of O^6^-alkylguanine position would result in a sterically hindered molecule that could no longer fit the MGMT substrate pocket.

### Toxicity of acrolein: ICLs and HOPdG

Acrolein is a highly reactive chemotherapeutic agent, generated by the cyclophosphamide class of nitrogen mustards (review [61]). Acrolein forms toxic DNA base adducts, notably α-HOPdG & γ-HOPdG. It also causes the formation of highly toxic DNA-protein crosslinks and interstrand cross-links (ICLs [61–66]), Figure 1B. Acrolein is extremely toxic in cells with DNA ICL repair defects, including cells derived from patients with Xeroderma pigmentosum (NER defects) [67] and Fanconi anemia (FA) [68–72].

Acrolein generated HOPdG lesions can be repaired directly via oxidation by three AlkBH enzymes, and indirectly by BER and MMR [61]. It has been shown that overexpression of ALKBH2 in glioma cell lines enhances resistance to TMZ, and conversely, siRNA-knockdown of ALKBH2 increases TMZ sensitivity [73]. Acrolein derived HOPdG is oxidized by ALKBH ∼200x slower than are DNA methyl adducts [74]. Thus co-administration of acrolein/TMZ (in a Stupp protocol chemotherapeutic regime) will impose an opportunity cost, whereby the time taken by AlkBH to oxidize a single HOPdG adduct means that 200 methyl adducts will not be removed by this direct mechanism [75, 76].

### The GBM157 model

All the *in vitro* and *in vivo* data presented herein derives from a de-identified human primary GBM. A GBM tumor was harvested from a patient and transplanted into mouse flank, and the resulting tumors were propagated and passaged in mouse flank and archived, frozen. Frozen tumor samples were then used to initiate flank tumors, which were then harvested and used to generate intracranial tumors. Cells from the original tumor were also used to generate tissue cultures, and these were archived, frozen, at 4–5 passages. The *in vitro* data that is presented using GBM157 are representative of experiments performed using other primary GBM cell lines.

GBM157 is one of the samples on our tumor microarray and the levels of MAOB are in the lowest quartile [45]. With regards to MGMT, we find total protein levels are in the highest quartile, as is HiF1-α, which is linked with MGMT expression levels [77]. We are currently further characterizing this line.

## RESULTS

### PAM-OBG: risk repair protein mRNA levels and patient survival in GBM

We examined the relationship between the mRNA levels of 181 DNA-repair genes and GBM patient survival using the data deposited in the TCGA database [2, 3]. This was to test the postulate that acrolein derived ICLs would be more toxic to GBM, than to non-cancerous cells, due to evolutionary pressures on glioma to down-regulate certain DNA repair functions.

Using the TCGA database (as of Dec 1st, 2017) we identified 110 (of 166) patients for which there was uncensored survival data (< 89 months) and whose treatment-naive tumors had been analyzed using RNA Seq V2. We examined the transcript levels of 181 genes, panned from the DNA repair pathway genes maintained by KEGG.org [26], involved in DNA repair. Survival curves were generated for each gene examined using ranked transcript data, comparing low and high mRNA levels and then assayed for statistical significance using the Cox-Mantel log-rank Chi-square test, (Mantel-Haenszel test). Raw and analyzed data is to be found in Supplementary Materials.

Of the 181 genes tested we found that 49 transcript levels impacted patient survival in a statistically significant manner (*p* < 0.05); 27 of these genes products (or pooled paralogues) were directly correlated with patient survival, (ALKBH1, ALKBH2, APEX1, APEX2, ASCC1, ASCC3, ATR, BLM, DCLRE1C, ERCC1, ERCC6, ERCC8, EXOG, GTF2H2C, MLH1, MPG, MSH4, MUTYH, NBN, PARP1&2, PMS1, POLDIP3, RAD23A, (RFC1-4), SLX1B, UBE2E1, UNG & XRCC5). Additionally, we identified 21 gene products (or pooled paralogues) where high levels correlated with poor patient survival, (ALKBH3, CHTF18, DMC1, HUS1, MGMT, MLH3, NEIL1, NEIL2, NEIL3, PAPD7, PARP3, PMS2, RAD17, RMI2, RPA3, RTEL1, SSBP1, TELO2, TERT, TOP3A, XPA & XRCC2).

In addition, we find that the PMS1/PMS2 and RAD23A/ERCC6 mRNA ratios, indicative of MutLβ/MutLα and of global/translation NER ratios respectively, to be independent risk factors in this GBM patient population.

Agnihotri and co-workers [39] found MPG to be a glioma risk factor, mainly examining pediatric tumors, and suggested that it could directly repair TMZ generated O^6^MeG lesions. We found that MPG expression also correlated with patient outcome, but that MPG effects were context dependent.

**Figure 2:**
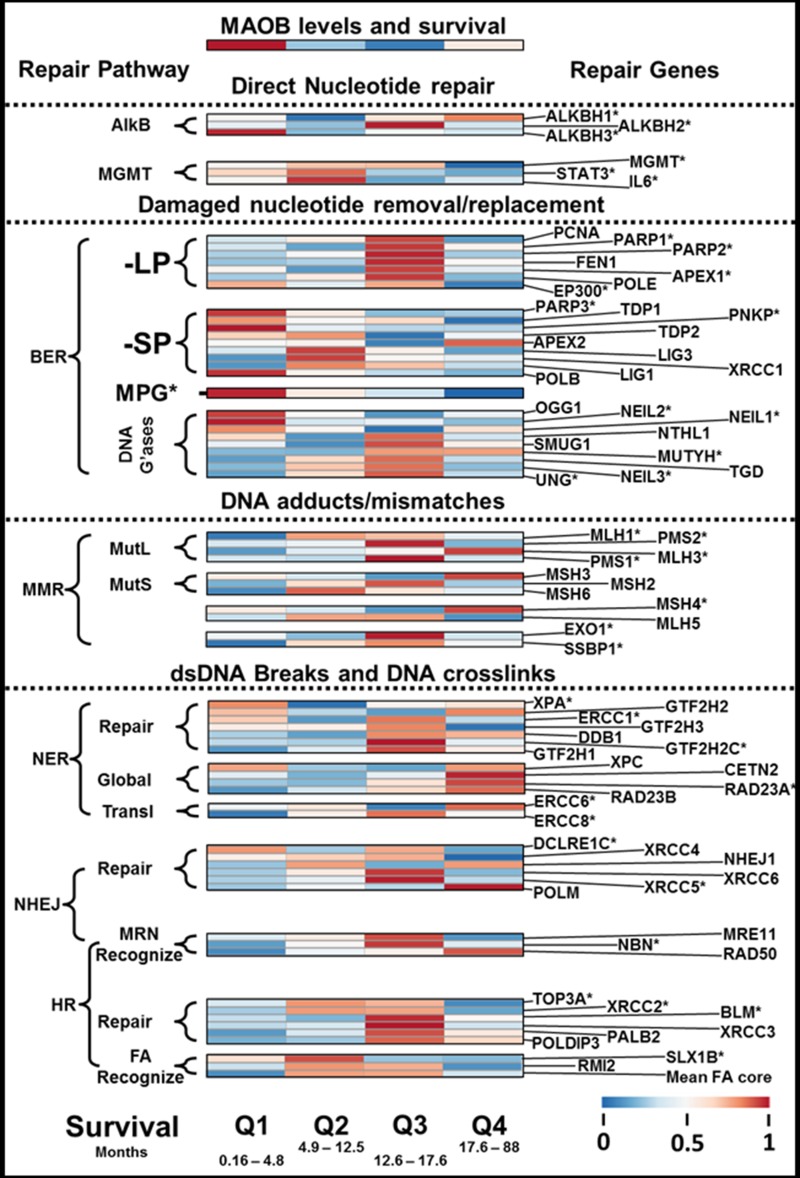
DNA repair enzyme transcript levels and GBM patient survival Figure 2 presents the levels of different DNA repair enzymes or pathways, in the form of a heat map, striated by GBM patient outcome. At the top of the heat map is shown the relative levels of MAOB, the PAM-OBG activating enzyme. It can be noted that the poorest responding patients, Q1, have the least sophisticated expression of DNA repair pathways that can repair dsDNA breaks or ILCs.

In Figure 2 we present a heat map of gene transcript levels, striated horizontally by patient survival outcome in quartiles and vertically by gene/function/pathway. The heat map was generated by taking the median mRNA level for each of the four patient groups. In Supplementary Figure 4 we show an additional heat map, also divided into survival quartiles, where we include the levels of transcripts for the 9-1-1 DNA repair pathway and telomere extension. Also included are the ratios of different core enzyme mRNA levels, which allow the ratios of repair pathways to be estimated in the different quartiles.

We will describe the influence of DNA repair enzymes on patient outcome, stratified by survival in quartiles, except for the case of MGT, which will be described separately.

### First quartile

The first quartile represents the patients with the worst outcome, and not reaching four and a half months survival after diagnosis. In these patients, we find the highest levels of MAOB and we also expected them to have high MGMT levels. However, these cells generally do not have high MGMT or STAT3/IL6. These tumors instead have high levels of another direct repair enzyme, ALKBH3. It has been shown by others that the pairing of ALKBH3 with a helicase (ASCC3) allows efficient removal of many dsDNA lesions, which are generated by TMZ, especially N^1^-MeA and N^3^-MeC [78]. In Q1 we find ALKBH3 paired with the helicase ASCC2, rather than the previously described ASCC3, and that the levels of ALKBH1, ALKBH2, ASCC1 and ASCC3 are low. In comparison with ALKBH1 and ALKBH2, ALKBH3 has far less non-repair functionality, as both ALKBH1 and 2 are involved in epigenetic modulation of tRNA, via demethylation of N^1^-methyladenosine RNA [79]. ALKBH1, but not ALKBH3, also demethylates histones, altering epigenetic gene expression [80]. ALKBH3 also lacks the potent AP-lyase activity found in ALKBH1 that can be pro-apoptotic [81].

High levels of the four DNA glycosylases, NEIL1, NEIL2, OGG1, and NTHL1 are found in the Q1 tumors, and these remove oxidized DNA bases and also TMZ generated N^7^-MeG and N^3^-MeA [82].

In Q1 tumors the main route of TMZ resistance appears to come from suppression of the canonical MMR pathway, halting futile cycling of O^6^MeG-DNA repair in DNA synthesis (see [83]), a well-reported mechanism of TMZ resistance [82, 84–87]. We find uniformly low levels of all MMR variants; MutLα, MutLβ and MutLγ, and also both MutSα and MutSβ, in this quartile.

Of the three major pathways that are capable of repairing dsDNA breaks, the NER pathway appears to predominate in these tumors. Moreover, global, rather than translational NER is suggested by the RAD23A/ERCC6 ratio. Low levels of the 9-1-1 complex proteins, including MUTYH1 [88], indicate that MUTYH1/CHK1/ATR dsDNA break sensor/signal pathway may not be operational in this cohort.

We find elevated levels of the canonical telomerase pathway genes; RTEL1, TELO2, and TERT expressed in this cohort, and it has been previously been shown that elevated levels of TERC, TERT and RTEL1, correlated with poor outcome in GBM [89].

### The expected effect of PAM-OBG in the first quartile

The pattern of DNA repair pathways indicates poorly functional BER and MMR pathways and that PAM-OBG derived HOPdG lesions will slow ALKBH3 removal of TMZ generated DNA-methyl lesions. The ability of these cells in this cohort to remove ICLs is quite limited, given the very low expression levels of the components of the Fanconi anemia core complex, components of HR, and components of NHEJ [70]. ICLs, induced by acrolein, can be removed at modest rates via NER, as we know that cells derived from patients with xeroderma pigmentosum, with dysfunctional NER, show sensitivity toward acrolein [67].

### The second and third quartiles

MGMT appears to be the main mechanism of TMZ resistance in Q2 and Q3, and the tumors in Q2 have high expression of STAT3/IL6, indicative of long MGMT biological half-life.

Short Patch BER (BER-SP) is DNA polymerase beta (POLB) dependent whereas PCNA-dependent Long Patch BER (BER-LP) is more promiscuous, using POLE, POLD or POLB [90]. The activity of POLB, also controlled by post-transcriptional modification and acylation by EP300, is high in Q3 and low in Q2, which essentially suppresses BER-SP but not BER-LP [91]. The difference in the usage of short/long patch BER in the two groups may lead to a tendency for evolutionary selection of XRCC1 based DNA repair, as POLB forms a complex with XRCC1 [92] or for PCNA based repair as POLE forms a tight complex with PCNA [93].

There is also a clear difference in the usage of MMR complexes, with the more aggressive Q2 GBM using MutLβ and MutLγ more than MutLα, and also MutSβ rather than MutSα. The canonical human MMR complex consists of MutSα (MSH2/MSH6) and MutLα (MLH1/PMS2), which is part of the ATR/CHK1 and ATM/CHK2 phosphorylation signaling system [94]. TMZ causes activation of the ATM and ATR kinases, phosphorylation of CHK1, CHK2, and p53, and triggering of the G(2)/M arrest via ATR/ATR MutSα/MutLα phosphorylation. Activation of ATM and of ATR has been implicated in the pro-apoptotic response to ionizing radiation. The lower MutSα/MutSβ ratio in Q3 compared to Q2, and loss of MSH6 signaling to ATR/CHK1 may explain these tumors sensitivity to chemoradiotherapy [95, 96]. In the Q1 cohort, NER appeared to be the major dsDNA break/ICL repair pathway, whereas in Q3 the GBM were biased toward NHEJ/MMEJ [97], and to HR, with FA core complex functionality [71]. Many of the tumors in Q2 lack major components of NER, HR and NHEJ/MMEJ pathways and may use hybrid repair process for eliminating ICLs and DNA double-strand breaks (DSBs).

### The expected effect of PAM-OBG in the second and third quartiles

The expression of MGMT, with STAT3 and IL6, indicates that direct repair is the main means of overcoming TMZ toxicity. The generation of O^6^BG, from PAM-OBG, in these tumors will cause sensitization via MGMT inhibition. The tumors reliant on BER-LP will be highly sensitive to MMR repair futile cycling of O^6^MeG [71].

Acrolein lesions can be recognized by FA and repaired by HR in competent cells, but these repairs are slow and cause replication stalls, making cells highly vulnerable to daily cycles of chemoradiation.

### The final quartile

These GBM have low MGMT and STAT3/IL6 levels explaining the better response to therapy. These tumors have poor BER and MMR pathway activity, and DSB/ICL repair appears to be a function of global, not translational, NER. They express some canonical NHEJ proteins but there is little evidence for FA core complex functionality.

### The expected effect of PAM-OBG in the final quartile

Denuding the low levels of MGMT expressed by these cells will further sensitize them to TMZ. Their ability to deal with acrolein lesions, especially ICLs, appears to be poor.

### MPG and patient outcome

In Supplementary Figure 5 we show three patient survival curves, with respect to MPG levels with Supplementary Figure 5A showing the whole patient population (*n* = 110). In the 71% of patients with low levels of MPG median survival is 11.8 months, the 29% of the population with high MPG have a median survival of 13.6 months, *p* = 0.023. This would appear to be in contradiction of the basic finding of Agnihotri and co-workers [39].

We stratified the patient population with respect to expression of XRCC1 (BER-SP), with one sub-population (*n* = 33) with high XRCC1 and the second sub-population (*n* = 77) with low XRCC1. We examined the effects of MPG in these two sub-populations.

In GBM with low BER-SP activity (low XRCC1 levels), MPG correlates with poor outcomes, with low MPG correlated to median survival of 15.2 months and high-level MPG to only 7.9 months, *p* = 0.02, Supplementary Figure 5B. In the subpopulation with high XRCC1 levels, MPG aided survival with patients with high MPG levels having a median survival of 14.1 months, but those with low MPG levels having a median survival of only 7.7 months, *p* = 0.015, Supplementary Figure 5C.

It, therefore, appears that MPG can remove toxic TMZ-adducts, including perhaps O^6^MeG, in cells utilizing BER-LP, whereas in GBM with efficient BER-SP, MPG sensitizes GBM to treatment. MPG toxicity in BER-SP, but not BER-LP, is most likely due to mitochondrial DNA repair directed apoptosis described by Fishel and co-workers [98]. It was shown that MPG repair of methyl lesions in mtDNA initiates mitochondrial driven apoptosis, in cases when the MPG glycosylase activity is higher than the downstream repair processes in BER-SP, causing an overload in AP-sites and DNA breaks.

### The expected effect of PAM-OBG on MPG-based DNA repair

MPG can excise some oxidative adducts, including some generated by acrolein [99]. MPG levels correlate with POLB, MGMT, and ALKBH3 Supplementary Materials, the former being complexed with XRCC1 in BER-SP and the latter pair directly removing adducts from DNA. In these cells, the generation of O^6^BG- and acrolein adducts, from PAM-OBG, will sensitize the tumor cells to chemoradiation.

### PAM-OBG sensitizes GBM cells toward alkylating agents *in vitro* via a MAOB mechanism

**Figure 3:**
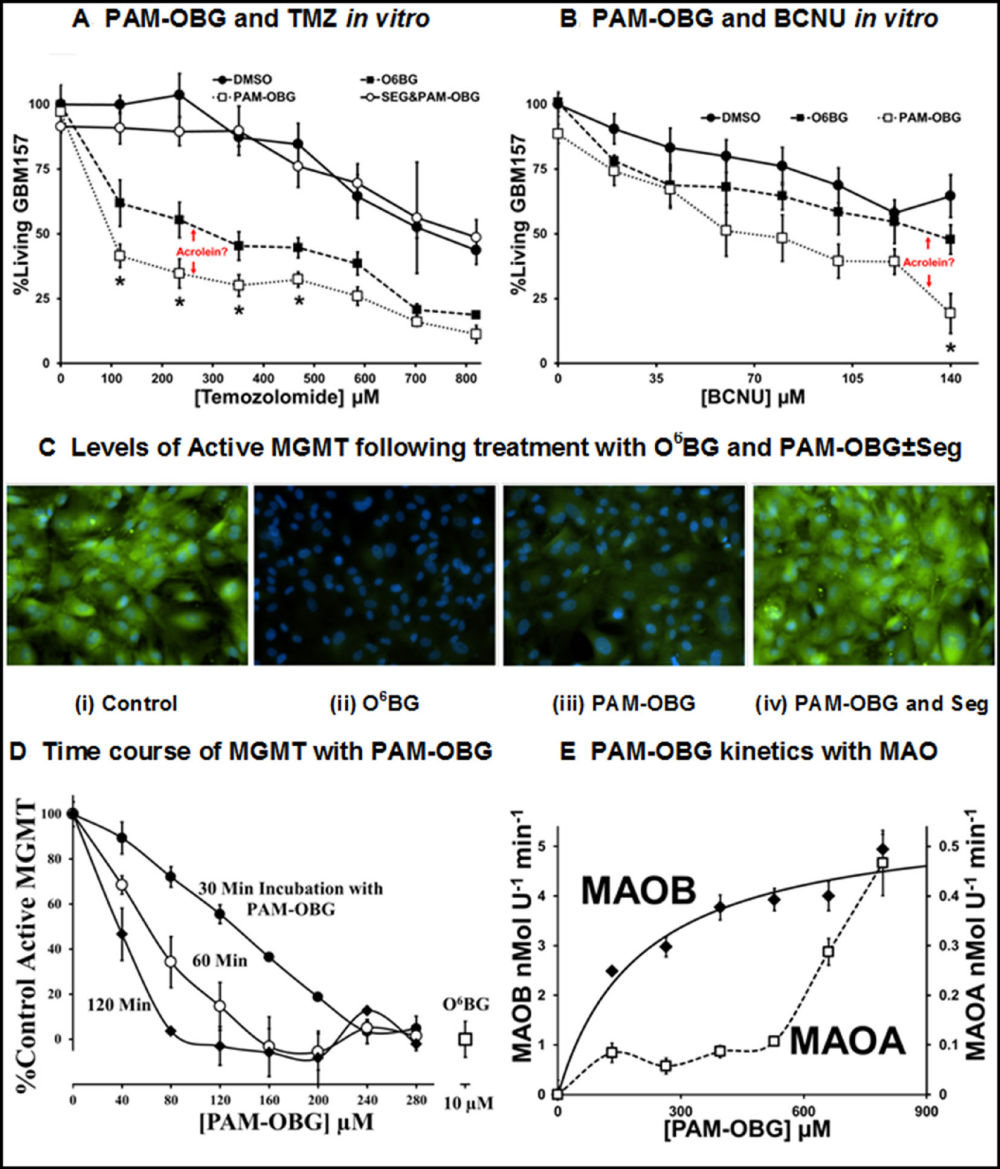
PAM-OBG is a MAOB sensitive prodrug (**A** and **B**) show the differential effects of O^6^BG and PAM-OBG, on the toxicity of TMZ (A) and BCNU (B), measured at 24 hours, *n* = 3 and 4 respectively, in primary GBM cell cultures. In both cases, PAM-OBG is a better potentiation agent than is O^6^BG. (A) also shows that the synergistic effect of co-administration of PAM-OBG and TMZ is MAOB dependent, as the addition of selegiline abolishes potentiation. The *t*-test was used to compare cell numbers of the O^6^BG and PAM-OBG treated cultures at each TMZ or BCNU concentration and those pairs that are statistically significantly different at the *p* < 0.05 level are identified with a ‘^*^’. (**C** and **D**) demonstrate that PAM-OBG depletes active MGMT levels, with active MGMT labeled by O^6^PGG and visualized by ‘clicking’ with azido-FITC. The green FITC signal seen in (C) is due to the presence of active MGMT in GBM cell cultures. Treatment with 10 µM O^6^BG (ii) or 100 µM PAM-OBG (iii) drops this fluorescence with respect to the control (i). The loss in signal MGMT caused to PAM-OBG can be abolished by inhibiting MAOB with a pre-incubation of 10 µM Selegiline (iv). The effect of incubation time and PAM-OBG concentration on the loss of active MGMT in cultured glioma, at *n* = 4, has the line-shape of an enzymatic transformation of PAM-OBG, (D). Using recombinant human MAOA or MAOB, *n* = 4, the kinetics of PAM-OBG oxidation show that PAM-OBG is a good MAOB substrate, and the fit indicates that the K_m_ is 200 µM for MAOB, but MAOA is a very poor substrate with a K_m_ of > 700 µM (**E**).

We titrated primary GBM cells, GBM157, with TMZ or BCNU in the presence and absence of PAM-OBG or O^6^BG, Figure 3A and 3B. GBM157 cells are resistant to both TMZ and BCNU, as they have high levels of MGMT and the IC_50_ values are ≈ 720 µM and > 150 µM, respectively.

In cells incubated with 10 µM O^6^BG, there was sensitization toward TMZ and BCNU, with IC_50_ values dropping to ≈ 280 µM and ≈ 130 µM, respectively. These data are consistent with the known MGMT drug resistance to TMZ and BCNU because TMZ is more efficient at generating O^6^G adducts than is BCNU [82, 100, 101].

We demonstrate that the sensitization observed in the presence of PAM-OBG is due to MAOB directed catalysis by pre-incubating cells with selegiline, a MAOB specific inhibitor, followed by PAM-OBG/TMZ. When MAOB is inhibited we observe no synergistic effect of PAM-OBG and either DNA alkylating agent.

PAM-OBG was a better sensitizer than was O^6^BG with both chemotherapeutics, with the IC_50_ falling to ≈ 30 µM for TMZ and to only ≈ 60 µM in the case of BCNU. We believe that this enhancement of toxicity observed with PAM-OBG vs. O^6^BG is due to the generation of acrolein from the MAOB catalytic oxidation of PAM-OBG.

### PAM-OBG only denudes glioma cells MGMT when MAOB is active

It has been demonstrated that ubiquitination, and subsequent proteolysis, of alkylated MGMT is more rapid than that of the native, active enzyme [102, 103]. In initial experiments, we incubated primary GBM157 cells in BCNU, O^6^BG, and PAM-OBG ± Seg and observed that PAM-OBG resulted in higher than canonical MGMT molecular weight bands, which we interpret as evidence for ubiquitination [103, 104]. Western Blots interrogating MGMT, as shown in Supplementary Figure 6, appear to show ubiquitinated high molecular weight bands as observed by Kuo and coworkers [105].

To better observe the alkylation and inactivation of MGMT by PAM-OBG we developed a method to determine the levels of active MGMT using a substrate, O^6^-propargyl-guanine (O^6^PGG) that is readily amiable to visualization by linking an azido-functionalized fluorophore via click-chemistry [106, 107]. O^6^-propargyl-guanine reacts with MGMT to generate a propargyl-thioether that can be covalently linked to azido-PEG-FITC by incubation with Cu(II), and therefore be visualized using fluorescence microscopy.

Primary glioma cells were incubated with an MGMT substrate, either O^6^BG or PAM-OBG, for a defined time period and then 100 µM O^6^PGG was added, and 10 minutes later the cells were fixed with paraformaldehyde (PFA). Following permeabilization and washing, thioether-propargyl MGMT was covalently linked to FITC-PEG-azide using Cu(II). Figure 3C (i) shows a representative cell field and the green fluorescence labels active MGMT, and blue DAPI signal, DNA.

Pre-incubation of cells with 10 µM O^6^BG before application of O^6^PGG causes (almost) complete loss of active MGMT signal, Figure 3C (ii). A 75% loss of active MGMT is observed with cells incubated with 100 µM PAM-OBG show, Figure 3C (iii). This MAOB dependent loss of active MGMT with PAM-OBG is demonstrated in Figure 3C (iv) where we show that co-incubation of MAOB inhibitor selegiline and PAM-OBG does not alter MGMT levels.

The relationship between PAM-OBG concentration, incubation time, and levels of active MGMT was explored by titrating glioma with PAM-OBG for 0.5, 1 and 2 hours, prior to addition of O^6^PGG. In Figure 3D we show the drop in (active) propargyl-MGMT levels as a result of PAM-OBG conversion to O^6^BG. The line-shapes resemble truncated and inverted Michaelis–Menten kinetic plots and suggest that the K_m_ for MAOB with PAM-OBG is between 160 and 200 µM (see 30-minute plot).

We confirmed this kinetic parameter using classical enzymology, titrating hrMAOA and hrMAOB with PAM-OBG and measuring the H_2_O_2_ generated from the reaction, via the HRP/Amplex Red method [56], Figure 3D. PAM-OBG is a good MAOB substrate but is a poor MAOA substrate. The data indicate that the K_m_ of MAOB for PAM-OBG is ≈ 200 µM and its V_max_ ≈ 13% of that observed with control MAOB substrate, benzylamine.

### Acrolein derived from PAM-OBG causes DNA damage

The role of NER in ICL repair is the subject of two reviews, the first by Wood (NER) [108] and the second by Williams, Gottesman, and Gautier (Global/Translation NER and overlap with NHEJ/HR/FA) [109]. CDK7 functionality is integral to translational NER repair, being a core component of the TFIIH complex, and the drug compound SNS-032 is a good inhibitor of this kinase [110–112].

In addition to inhibiting CDK7, SNS-032 also inhibits the cyclin-dependent kinases CDK2, CDK5 and CDK9 [113], however, even though SNS-032 can alter glioma response to hypoxia, it is not toxic toward U87 cells even at very high drug concentrations (500 nM) [114].

In primary acute myeloid leukemia (ALM) SNS-032 acts in a synergistic fashion toward the deoxycytidine memetic drug Cytarabine [115], and it is also known that AML sensitivity, and patient outcome with cytarabine therapy, is linked to polymorphisms in nucleotide excision repair genes [116]. When CDK7 siRNA is used in cells it is found that translational NER is essentially halted, and the cells are sensitized to *UV*-induced interstrand crosslinks [117]. Translational NER is the main pathway used in the repair of cisplatin-induced ICLs [118], and treatment of cells with both CDK7 siRNA and cisplatin results in a 14-fold increase in toxicity [119].

**Figure 4:**
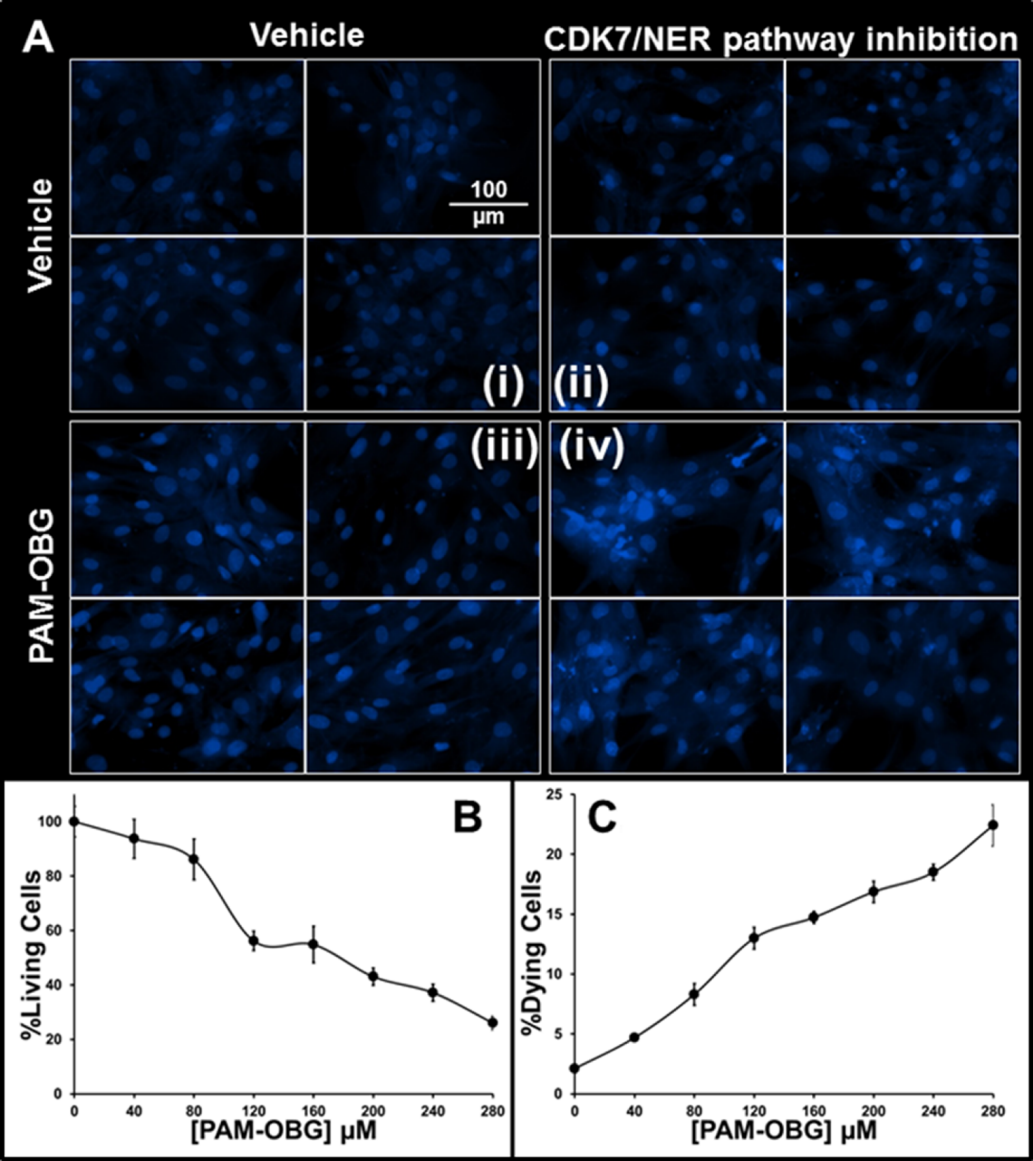
PAM-OBG derived acrolein is toxic in SNS-032 treated cells (**A**) shows that acrolein derived from PAM-OBG is DNA damaging and these lesions are normally removed by a CDK7-sensitive NER repair process. Cells were incubated with/without 100 nM SNS-032, an inhibitor of CDK7, to an *n* = 6, and titrated with PAM-OBG, and labeled with Hoechst 33342 viability dye. In (A) we show four panels of high-resolution images of glioma cell nuclei in control cells (i), cells incubated with SNS-032 (ii), with 280 µM PAM-OBG (iii) and SNS-032 with PAM-OBG (iv). Only in glioma incubated for 24 hours with both PAM-OBG and the CDK7 inhibitor do we observe apoptotic cell nuclei and fragmented nuclei that are diagnostic of mitotic catastrophe. The fall in viable cells numbers and increase in dying cells 24 hours after treating GBM157 cells with SNS-032 and PAM-OBG is shown in (**B** and **C**), respectively.

The potential synergistic effects of PAM-OBG with SNS-032 was examined to determine if acrolein dependent ICLs are generated by the action of MAOB on PAM-OBG. The growth of GBM157 treated with 100 nM SNS-032 slowed by 17% over 24 hours (*p* < 0.024), but there was no noticeable effect on cell death; see DAPI nuclei in Figure 4A (i) and (ii). GBM157 cells incubated for 24 hours in 280 µM PAM-OBG showed a 10% drop in cell numbers (*p* < 0.01) and again there was no increase in cell death rates, Figure 4A (iii). Co-incubation of SNS-032 with PAM-OBG is devastating; we observed a 75% drop in viable cell numbers and the nuclei in surviving cells show abnormal structures and evidence of mitotic catastrophe [120, 121], Figure 4A (iv).

In Figure 4B and 4C, we show the viable cells numbers and levels of dead/dying observed in cells treated with SNS-032 and titrated with PAM-OBG. PAM-OBG is highly toxic in cells with NER pathway inhibition. As we know that PAM-OBG generates acrolein, and we also know that acrolein generates ICLs and that the NER DNA repair pathway is sensitive toward SNS-032, it is reasonable to conclude that in glioma cells PAM-OBG gains part of its toxicity from acrolein-induced DNA-adducts.

### Sensitizing intracranial primary GBM tumors to BCNU and CCNU with PAM-OBG

**Figure 5:**
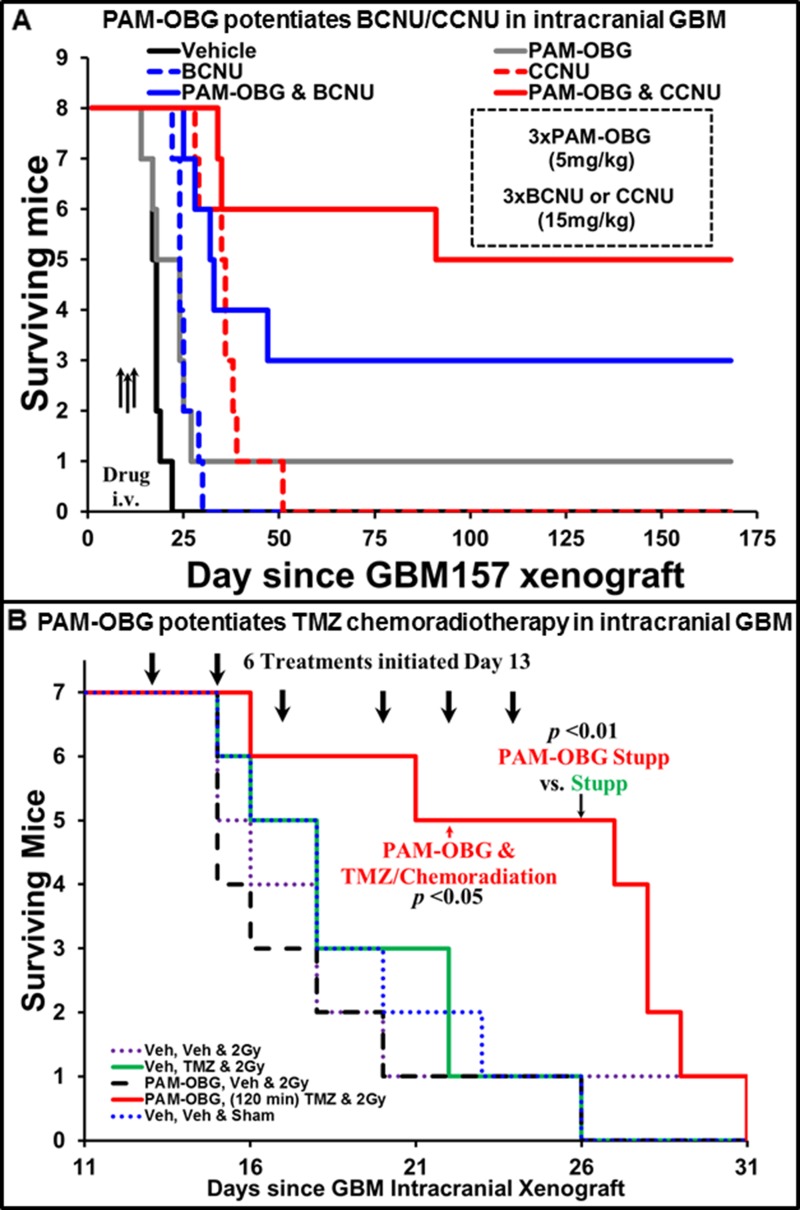
PAM-OBG potentiates the treatment of intracranial GBM tumors in mice Figure 5 reports the survival curves are two studies where mice with intracranial GBM157 tumors were treated with PAM-OBG. (**A**) shows that in addition to potentiating the anti-cancer activity of BCNU and CCNU, it can increase survival of tumor-bearing animals as a mono-agent. Analysis of the survival curves shows that PAM-OBG increases survival of the animals by 6-fold, compared with alkylating agent monotherapy. (**B**) shows the effectiveness of PAM-OBG in a TMZ chemoradiotherapy intracranial mouse model. Beginning on day 13, post-xenograft, mice were given six rounds of intravenous PAM-OBG, TMZ by gavage two hours later, and then an hour later, 2Gy whole head radiation. This combined therapy gave a half-survival time of 27.5 days, more than 8 days more than animals who received only TMZ/2Gy irradiation. This highly aggressive GBM was unresponsive to treatment with radiation, PAM-OBG as monotherapy or to TMZ and 2Gy, with all control groups being statistically identical.

In Figure 5A we show animal survival curves of mice with intracranial GBM157 xenografts treated with BCNU or CCNU with/without PAM-OBG.

Control animals received two tail vein injections of vehicle, two hours apart, on days 10, 12 and 14 post-xenograft. This cohort reached an ethical end-point at a median time of 16.8 ± 1.6 days. Mice that were given PAM-OBG (5 mg/Kg) performed better, reaching a median survival time of 22.17 ± 4.85 days, and one animal was symptom-free, and healthy, for the full length of the 170-day study. Three intravenous injections of 15 mg/Kg of BCNU or CCNU were able to extend life some 7 and 18 days beyond the vehicle controls, with the median ethical end-points being reached at 24 ± 2.5 and 35 ± 4.8 days respectively. If the survival enhancement of combining PAM-OBG with the alkylating agent was additive we would expect that mice treated with BCNU/PAM-OBG to have a survival midpoint of 29.2 ± 6.7 days, yet the cohort that received this treatment had a 50% survival value of 92 ± 44 days. CCNU in combination with PAM-OBG proved to perform much better than the additive 39.9 ± 9.2 day midpoint, as this combination gives a value of 152.6 ± 64 days for 50% animal survival.

In addition to improving survival the combination of BCNU/CCNU with PAM-OBG in these mice we noted general health indicators, pink eyes, highly active behavior during handling, and they also put on weight, gaining ≈ 4.5 and 6 grams respectively.

### Sensitizing intracranial primary GBM tumors to TMZ/radiation with PAM-OBG

Patients are of course typically treated with TMZ and radiation, and not BCNU or CCNU, and we wished to use PAM-OBG with chemoradiation in a mouse intracranial GBM xenograft model.

Without knowing the pharmacokinetics of PAM-OBG in this model we carried out a provisional study where we varied the time between intravenous PAM-OBG injection and receipt of TMZ by gavage, followed by whole head radiation an hour later. As we did not know the effectiveness of individual therapies (radiation, TMZ and PAM-OBG), we used a low GBM inoculum in the xenograft and treated the animals early after this procedure, on days 8, 10 and 12. This was done to stagger mouse survival between the groups by allowing treatments to have high efficacy on the model.

The survival curves of this initial study are shown in Supplementary Figure 7 and clearly demonstrated that the optimum time between injecting PAM-OBG and TMZ gavage was between 90 and 150 minutes. The study was halted after 170 days due to two animals reaching an ethical end-point unrelated to the xenograft (two of the mice had ulcerated bite wounds).

In Figure 5B we show the results of PAM-OBG on chemoradiotherapy, in a ‘mini-Stupp’ protocol.

We deliberately used a late treatment intracranial model, where the animals have a high tumor burden before treatment, in an attempt to recapture the conditions found in GBM patients who have very poor outcomes. In this model, we initiated therapy four days before the typical median survival of untreated mice. For the study, we required 35 animals, in 5 groups of 7 animals (the number of slots in our mouse-body radiation shield). However, we gave 50 mice primary GBM157 xenografts, a 43% excess because we knew a large fraction would reach an ethical end-point prior to the start of treatment. On day 13, when we randomized the heaviest 35 mice into five treatment groups of *n* = 7 animals, 11 mice (22%) had arrived at an ethical end-point.

Treatment was given three days a week (M/W/F), for two weeks, but mice were examined daily throughout the study. In this ‘mini-Stupp’ model the treatment group of mice received 5 mg/Kg intravenous PAM-OBG, two hours later they were given 10 mg/Kg of TMZ by gavage, and then one hour later they received 2Gy of whole head radiation. There were four groups of control animals, the vehicle group received Veh/Veh/sham irradiation and reached a median lifespan of 18.7 ± 3.8 days. The radiation group, Veh/Veh/2Gy had a survival mid-point of only 17.2 ± 3 days. In mice given six treatments of TMZ chemoradiation, Veh/TMZ/2Gy, survival was 18.9 ± 3.9 days. PAM-OBG monotherapy indicated that PAM-OBG is not a radiosensitizer as giving PAM-OBG three hours before radiation, resulting in a median lifespan of only 16.7 ± 3.2 days. There was no statistically significant difference in the survival curves of any of the four control cohorts. In the treated group, PAM-OBG/TMZ/2Gy, the mid-point survival was very much extended, reaching 27.5 ± 7.79 days, and this group achieved statistical significance, with respect to the chemoradiation group on day 22 at *p* < 0.05 and day 26 the *p*-value fell below 0.01, as on day 26 there were no survivors in the Veh/TMZ/2Gy group, but 70% of the treatment group were alive.

## DISCUSSION

An extensive analysis of mRNA expression of different components of DNA repair pathways and GBM patient outcome indicates defects in the ability of aggressive GBM to repair ICLs. Our analysis supports the postulate that GBMs have evolved the ability to rapidly evolve by downregulating some DNA repair pathways so as to increase genomic instability and mutation rates. A loss in DNA replication fidelity, due to downregulation of DNA repair pathways as a general feature in cancer has been postulated by others [122–124]. The most aggressive and lethal GBM’s are characterized by having high levels of ALKBH3, low levels of canonical MMR and appear to rely on NER for the repair of dsDNA breaks and the removal of ICLs. In patients that succumb to GBM between 5–12 months MGMT, perhaps potentiated by STAT3 phosphorylation increases in pre-treatment MGMT steady-state levels, and short patch BER appear to the main means of TMZ chemotherapeutic resistance. Patients who survive between 12.5 and 18 months tend to have relatively high levels of MGMT, but no STAT3 potentiation, and are characterized by having high MMR levels. Those patients who survive longer than 18 months are characterized as having low MGMT levels, fully functional MMR and a wide range of repair systems for dsDNA breaks and ICLs.

PAM-OBG is a MAOB specific prodrug that generates O^6^BG and acrolein after enzymatic oxidation. MAOB levels are higher in low-grade glioma and in GBM than in the body’s other tissues. Thus PAM-OBG can ablate MGMT activity and create acrolein-DNA adducts in glioma cells, but not in tissues with low levels of MAOB. We have demonstrated that PAM-OBG is able to generate O^6^BG inside glioma, via MAOB, and that this O^6^BG then reacts with MGMT inactivating it. PAM-OBG treatment of glioma sensitizes cells to alkylating agents *in vitro* and *in vivo*. The acrolein generated by MAOB catalyzed immolation of PAM-OBG causes DNA damage and these acrolein derived lesions are highly toxic. Acrolein generated by PAM-OBG potentiates the toxicities of alkylating agents BCNU and TMZ and we demonstrate that the acrolein generated ICLs are normally repaired by the glioma translational NER pathway.

PAM-OBG ablates glioma MGMT in mouse intracranial models, potentiating TMZ chemoradiation, however, PAM-OBG does not itself potentiate radiotherapy. The increased survival of animals treated with PAM-OBG and TMZ/radiation is due to the generation of O^6^BG, inhibiting MGMT, increasing the steady state level of TMZ-induced O^6^MeG in DNA and also to the formation of acrolein adducts.

We believe that subject to the results of toxicity studies, that PAM-OBG could aid the treatment of GBM patients as an addition to current best treatment, the Stupp protocol. In addition, PAM-OBG may be useful in salvage therapy when used in combination with β-chloro-nitrosoureas like BCNU and CCNU or cisplatin-based PVC therapy.

## MATERIALS AND METHODS

### Materials

Unless otherwise indicated reagents were sourced from Sigma-Aldrich (Sigma-Aldrich, St. Louis, MO, USA). Mice were obtained from Charles River Laboratories, Inc. (Wilmington, MA, USA).

### Human rights and informed consent

All procedures followed were in accordance with the ethical standards of the responsible committee on human experimentation (institutional and national) and with the Helsinki Declaration of 1964 and later versions. Informed consent or substitute for it was obtained from all patients for being included in the study.

### Permission

The animal research in mice was conducted according to Institutional Animal Care and Use Committee (IACUC) Protocol AUP-0315-0016, approved by the IACUC of Methodist Hospital. All animal care procedures conformed to the Guide for the Care and Use of Laboratory Animals (National Research Council, National Academy Press, Washington DC 1996, USA). Glioma tumors and resulting cultures used were from de-identified patient tissues and have no identifiable private information under IRB Protocol 00014547.

### Tumor samples

A GBM tumor was taken at the time of excision and given the laboratory ID of GBM157. It was washed in Phosphate Buffered Saline (PBS, Fisher Scientific, Waltham, MA), chopped with a scalpel and homogenized in a BeadBug™ homogenizer (Benchmark Scientific, Inc. Edison, NJ) using 1.5 mm Zirconium beads, in an equal volume of PBS. An aliquot was diluted 1:1 in Matrigel™ and injected into the flanks of two Balb/c mice. Tumors were passaged twice in Balb/c mice and then expanded in nude mice (NU-Foxn1^Nu^) for three passages, harvested, frozen and archived.

### Intracranial models

For the generation of GBM intracranial xenografts flank tumors were resurrected from frozen stocks and passaged in nude mice (NU-Foxn1^Nu^) twice and a donor tumor was harvested when the volume was approximately 1.5 cm^3^. The tumor was homogenized using a BeadBug™ homogenizer, in an equal volume of PBS and then diluted 1:1 in Matrigel™ and mice were given intracranial xenografts with 5 µl percutaneous injections directly into the brain via the postglenoid foramen [57, 125, 126].

PAM-OBG and BCNU and CCNU were given as intravenous injections of 100 µl using 20% Kolliphor^®^ EL emulsification agent. TMZ was given as 100 µl oral dose dissolved in commercial gavage solution.

A pair of brain tissue sections, labeled with H&E or with DAP/V9 antibody, taken from a control animal with an intercranial xenograft is shown in Supplementary Figure 8.

### Irradiation

We used a modification of the mouse irradiation holding system demonstrated by Grasso and co-workers [127]. Mice were anesthetized using an Isoflurane chamber and were then given 1 mg/kg of the liquid anesthetic Dexmedetomidine. Mice were transferred into 50 ml Conical Centrifuge Tubes, which had had the ends cut off, allowing their noses to protrude from the conical ends. Domestic pipe-lagging expanded polyurethane insulation was custom cut to hold the mice in position. Seven mice were placed into a pre-warmed (37°C), custom-made radiation-shield, which has seven ports to hold the mice in centrifuge tubes, with their heads above the shield. The shield housing was made from hollow aluminum centrifuge tube holder and the inner void is filled with nickel-coated lead bird shot. This design of shield was chosen as it can be fabricated in workshops that lack the environmental protection measures against aerosol lead particles. The shield containing the mice was placed in the pre-calibrated, irradiation chamber ‘safe’ on its turntable and irradiated with 2Gy radiation from a cesium source rod.

After irradiation the mice were removed from the tube, given 1 mg/Kg Antisedan (Dexmedetomidine antidote), and placed in cages, lined with paper towels, sitting on heating pads (37°C). They were allowed to gradually come to, before being transferred into their cages.

### Cultured cells

Cells from the second aliquot of GBM157 homogenate were grown in Dulbecco’s modified Eagle’s medium (DMEM) with fetal bovine serum (FBS, 20%), 1U GlutaMax™, sodium pyruvate (1 mM), penicillin (100 U/ml), and streptomycin (100 mg/ml).GBM157cells are spontaneously immortal and were frozen at the fourth passage and used between seventh and ninth passages.

Glioma cells were grown to achieve confluency 24 hours after drug/vehicle dosing in Costar 96-well plates (Corning, NYC, NY, USA).

### Fluorescence microscopy

Images were captured using a Nikon Eclipse TE2000-E at 4×, 20× or 30× magnification using a CoolSnap ES digital camera system (Roper Scientific) containing a CCD-1300-Y/HS 1392-1040 imaging array that is cooled by Peltier. Images were recorded and analyzed using Nikon NIS-Elements software (Elements 3.22.11). All images were saved as JPEG2000 files using Nikon NIS-Elements. The emission of FITC-labeled MGMT was collected using ex 450–490 nm, em 500–550 nm and Hoechst 33342 using ex 325–375 nm, em 435–485 nm.

### Cell viability

Cells were incubated for 30 minutes with Hoechst 33342 (10 µM) and then were fixed with ice-cold 4% paraformaldehyde. We conducted cell counts in center field at ×4 or ×20 magnification, depending on cell density. Dead/dying cells were identified as having condensed nuclei with signal intensities over threefold that of the median cell nuclei or being identified as fragmented.

### Measurement of active MGMT levels

Active MGMT was labeled by incubating cells with 100 µM O^6^PGG for 10 minutes, prior to fixation. The copper-catalyzed azide-alkyne cycloaddition was performed in 100 µM CuSO_4_, 500 µM THPTA ligand, 5 mM ascorbate and 10 µM FITC-azido PEG, typically overnight at room temperature [128].

### Measurements of MAO activity by H_2_O_2_ generation

Amplex Red (150 mM), and HRP (3 U/ml) were incubated with rhMAOs (1 U/ml, Sigma) in pH 7.4 buffer (50 mM KPi, 0.015% lauryl maltoside) at 37°C for 15 min in 96-well format (75 mL volume per well) [56]. The formation of fluorescent resorufin (ex 500–560 nm, em 565–625 nm) was measured in a BioTeck Synergy HT spectrophotometer. The maximal signal generated from Amplex Red (150 nmol/ml) was established by the addition of H_2_O_2_ (1 mM) to each well at the end of the assay period.

### Synthesis

#### O^6^-propargylguanine

O^6^-Propargylguanine was synthesized as reported by Griffin and colleagues [129]. Propargyl alcohol (1.2 mmol) was dissolved in anhydrous THF and cooled to 0°C. A solution of sodium hydride (2 mol equiv) in dry THF was added and stirred for 30 min at 0°C. 2-Amino-6-chloropurine (1 mol equiv) was added and the mixture was heated to reflux under N_2_ until TLC analysis confirmed consumption of starting material. The reaction mixture was neutralized with glacial acetic acid, and the solvent was removed *in vacuo*. The product was purified by chromatography on silica (EtOAc: EtOH 9:1) and recrystallization from ethanol.

### FITC-azido-PEG

For ease of purification we used amino-PEG_5000_-azide (Sigma Cat#JKA5239) as our starting material for an azido-clickable fluorophore, as the product can easily be separated from starting materials. Amino-PEG_5000_-azide trifluoroacetate salt was added to fluorescein isothiocyanate in *N,N-*diisopropyl ethylamine (200 µL) and 5 ml anhydrous DMF, then stirred overnight. The resulting dark orange reaction mixture was dialyzed against 500 ml deionized water (MW_CO_ = 0.5–1 kDa), then further concentrated and washed by ultrafiltration (Millipore filter packs, MW_CO_ = 3000), centrifuged at 4000 rpm for 6 h.

### PAM-OBG

We will publish the synthetic route used and the purification methodology in a future manuscript. Briefly, commercial O^6^BG is purified by crystallization in ethanol and the N^9^-secondary amine is protected with chloromethyl pivalate. Aminopropanol was protected using trifluoroacetate. Triphosgene is used to couple the two, via a carbamate, and deprotection of both the pivalate and trifluoroacetate was performed by hydrolysis in 0.1M NaOH. The crude PAM-OBG was then purified by HPLC/recrystallization giving > 2-gram quantities of pharmaceutical grade.

## SUPPLEMENTARY MATERIALS FIGURES



## Abbreviations

(ALKBH): Alkylation repair homologs
(PAM-OBG): aminopropyl O^6^-benzylguanine N - carbamate
(ATM): Ataxia Telangiectasia Mutated Serine/Threonine Kinase
(BER): Base Excision Repair
(CDK7): Cyclin-dependent kinase 7
(ICL): DNA interstrand cross-link
(MGMT): DNA methyltransferase
(DSB): DNA double strand breaks
(FA): Fanconi anaemia pathway
(FITC): Fluorescein isothiocyanate
(GBM): glioblastoma multiforme
(HR): Homologous Recombination
(α-HOPdG & γ-HOPdG): α/β hydroxy-1, N^2^-cyclic propano-2′-deoxyguanosine
(IDH): Isocitrate Dehydrogenase
(BER-LP): Long Patch BER
(LGG): low-grade glioma
(MMR): mismatch repair
(MAO): Monoamine Oxidase
(MPG): N-Methylpurine DNA Glycosylase
(NHEJ): Non-Homologous End-Joining
(NER): Nucleotide Excision Repair
(O^6^BG): O^6^-benzylguanine
(O^6^MeG): O^6^-methylguanine
(O^6^PGG): O^6^-propargyl-guanine
(PCV): Procarbazine, CCNU and Vincristine therapy
(ROS): reactive oxygen species
(Seg): Selegiline
(Sham): Sham irradiation
(BER-SP): Short Patch BER
(TMZ): Temozolomide
(THPTA): Tris (3-hydroxypropyltriazolyl-methyl)amine
(Veh): Vehicle
