# Supplementary material for: PAM-OBG: A monoamine oxidase B specific prodrug that inhibits MGMT and generates DNA interstrand crosslinks, potentiating temozolomide and chemoradiation therapy in intracranial glioblastoma
Source: Oncotarget. 2018 May 8;9(35):23923–43. doi: 10.18632/oncotarget.25246 (PMC5963626; doi:10.18632/oncotarget.25246)
Supplement: Supplementary file 1 [file oncotarget-09-23923-s001.pdf]

PAM-OBG: A monoamine oxidase B specific prodrug that inhibits MGMT and generates DNA interstrand crosslinks, potentiating temozolomide and chemoradiation therapy in intracranial glioblastoma

SUPPLEMENTARY MATERIALS

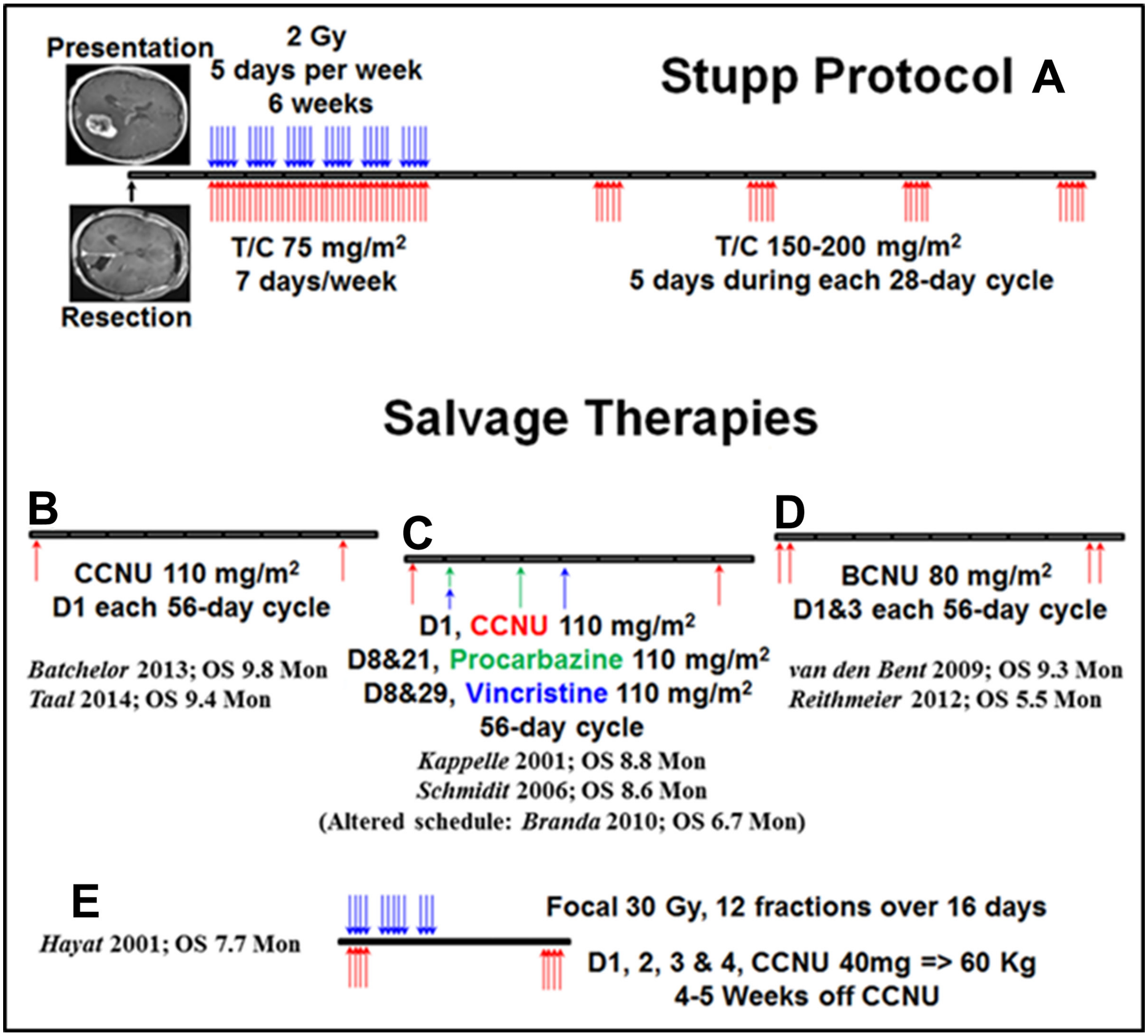

Supplementary Figure 1: The Stupp protocol and potential salvage therapies. In Supplementary Figure 1 we show standard care for GBM, 'the Stupp protocol' (A) and four different salvage therapy regimes, which PAM-OBG may aid, that have been used on the failure of TMZ maintenance therapy.

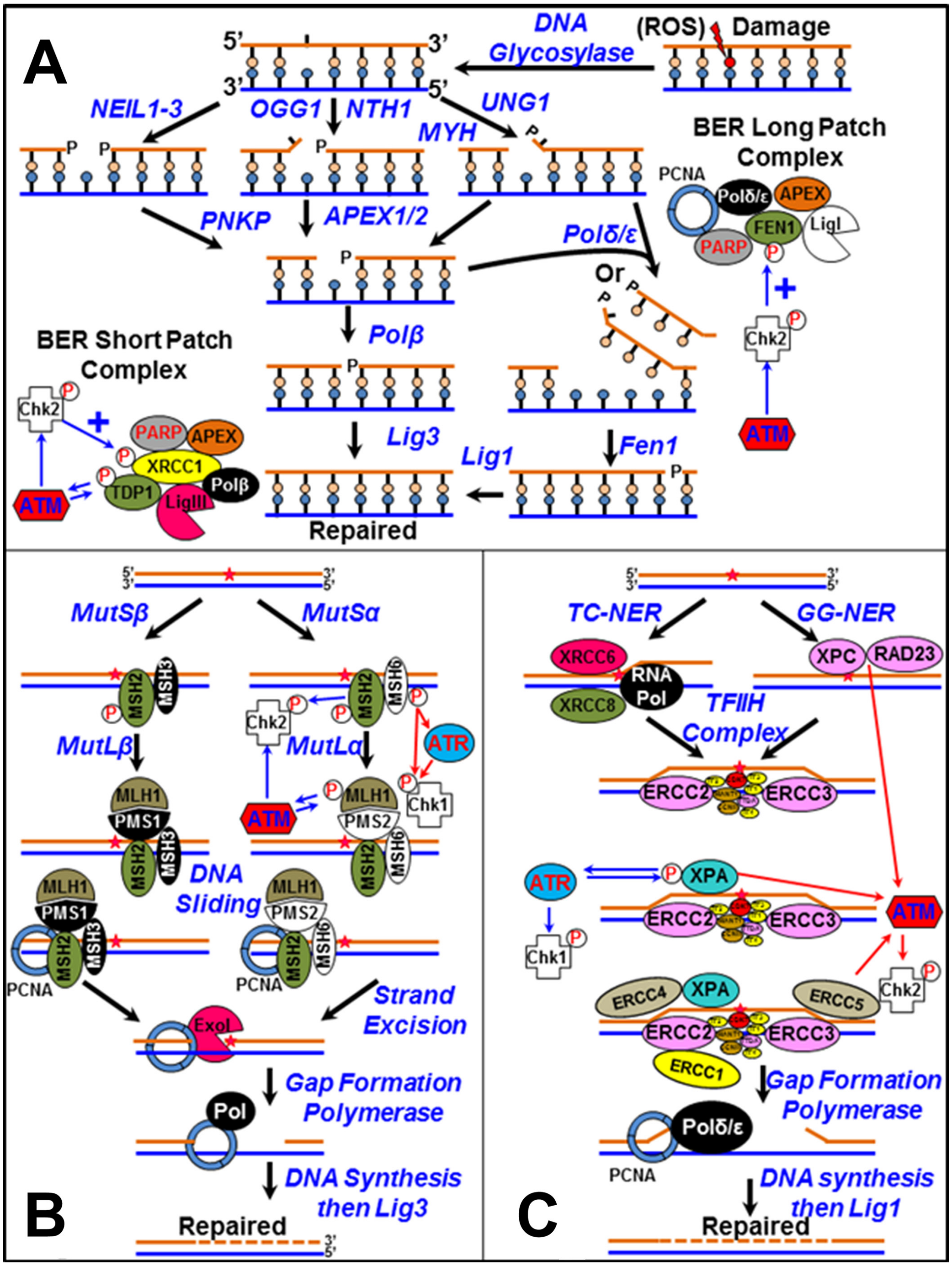

Supplementary Figure 2: BER, MMR, and NER DNA repair pathways. Supplementary Figure 2 outlines the mechanisms and proteins involved in both short patch and in long patch Base Excision Repair (BER) (**A**), Mismatch Repair (MMR) (**B**) and Nucleotide Excision Repair (NER) (**C**). BER: DNA glycosylases remove damaged bases giving rise to apurinic/apyrimidinic sites or DNA nicks. These AP-sites or nicks are sculpted to generate a DNA nick with 3’OH and 5’PO4 ends. In short patch BER, POLB and then XRCC1/LIG3 co-operate to insert an appropriate base and ligate the strand. In long patch BER, following clamping by PCNA either POLD or POLE (or POLQ) insert a number of complementary bases, 2–10 nucleotides, from the 3’OH end of the nick, creating a flap. The flap is removed by FEN1 and the nick ligated by LIG1. Both BER-SP and BER-LP are tied into the ATP/CHK2 reporting phosphorylation system. MMR: A mispair is recognized by a MutS heterodimer, causing MutL/PCNA recruitment and MutL nicks the newly synthesized strand next to the mismatch. In the Exo1-dependent subpathway, Exo1 excises the strand to a position past the mispair, which it is infilled and ligated by POLB/LIG3. In the absence of Exo1, POLD/E and FEN1 synthesize a complementary strand, past the mispair, the flap is excised and the LIG1 completes the repair. Canonical MMR MutSα/MutLα is in communication with ATM/CHK2 and ATR/CHK1 signaling systems, but this communication may be suppressed with MutSβ and with MutLβ and MutLγ. NER: XPC/R23B senses DNA helix-distorting lesions in global NER (GG–NER). In transcription-coupled repair (TC–NER) lesions are detected by RNA Pol blockage. The detection complexes communicate with ATR/ATM and attract the transcription factor IIH (TFIIH), a multicomponent complex composed of 10 subunits including CDK7. TFIIH opens a 10–20 nucleotide ssDNA bubble around the lesion using helicases XPB and XPD. XPA and RPA’s stabilize the ssDNA and XPG makes an incision on the 3′ of the lesion and ERCC1/XPF makes a second incision 5′ of the lesion. The 3′ end is clamped by PCNA, allowing the gap to be filled by POLD/E and ligation by LIG1.

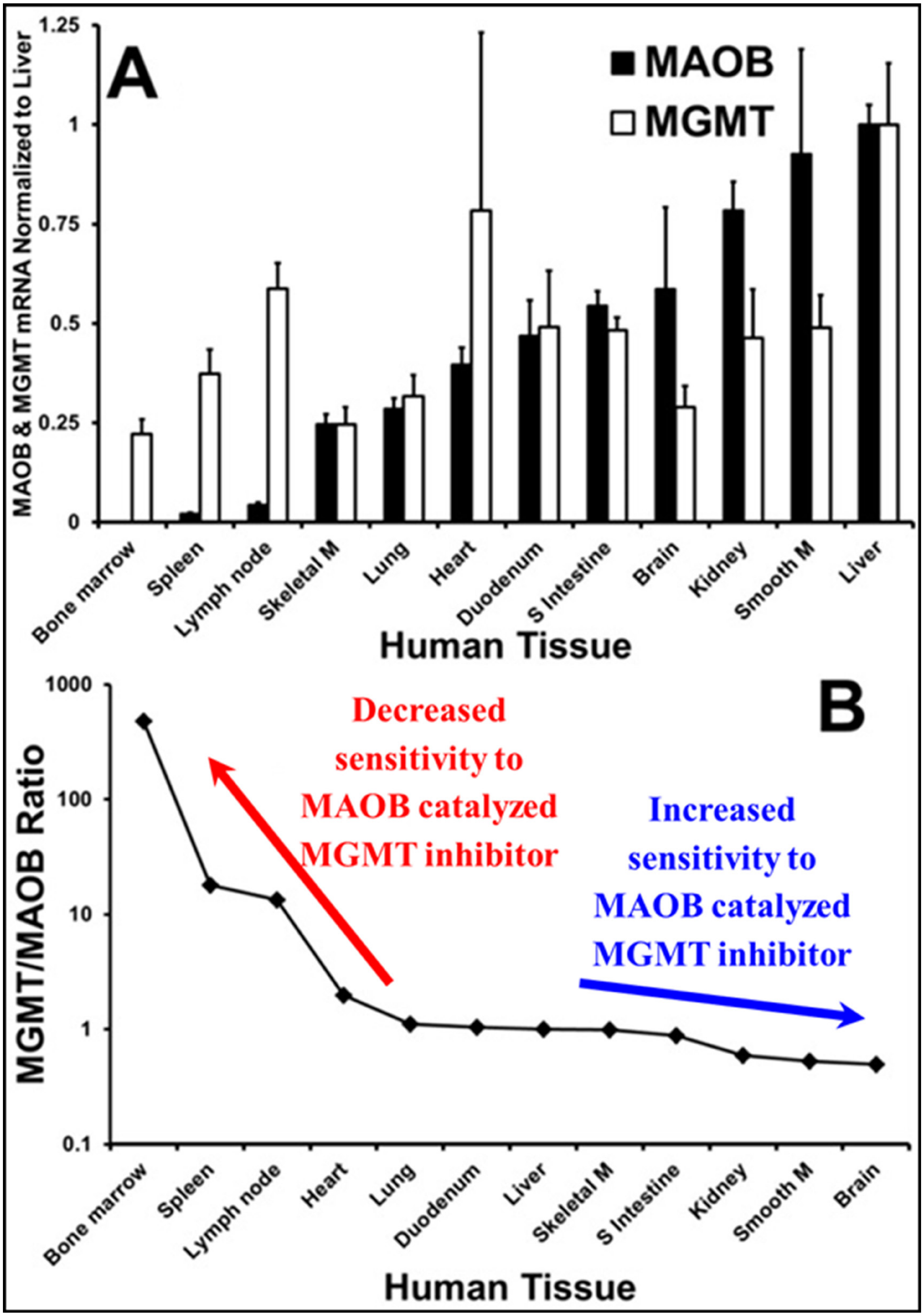

Supplementary Figure 3: MAOB and MGMT mRNA distribution in human tissues. Supplementary Figure 3 shows the levels of MAOB and MGMT in human tissues. (**A**) shows the levels of mRNA of MAOB and MGMT, normalized to the liver, in potential off-target human tissues and show that liver has the highest level of MAOB and MGMT transcripts and that MAOB is undetectable in bone marrow, a tissue with low MGMT levels and the typical limiting tissue in alkylating agent chemotherapy. (**B**) presents the same data in the form of the MGMT/MAOB ratio. Tissues with high MAOB and low MGMT will be most sensitive to a PAM-OBG type prodrug and in humans, this would appear to be the brain, but human bone marrow appears to be the least sensitive to a MAOB-sensitive anti-MGMT prodrug.

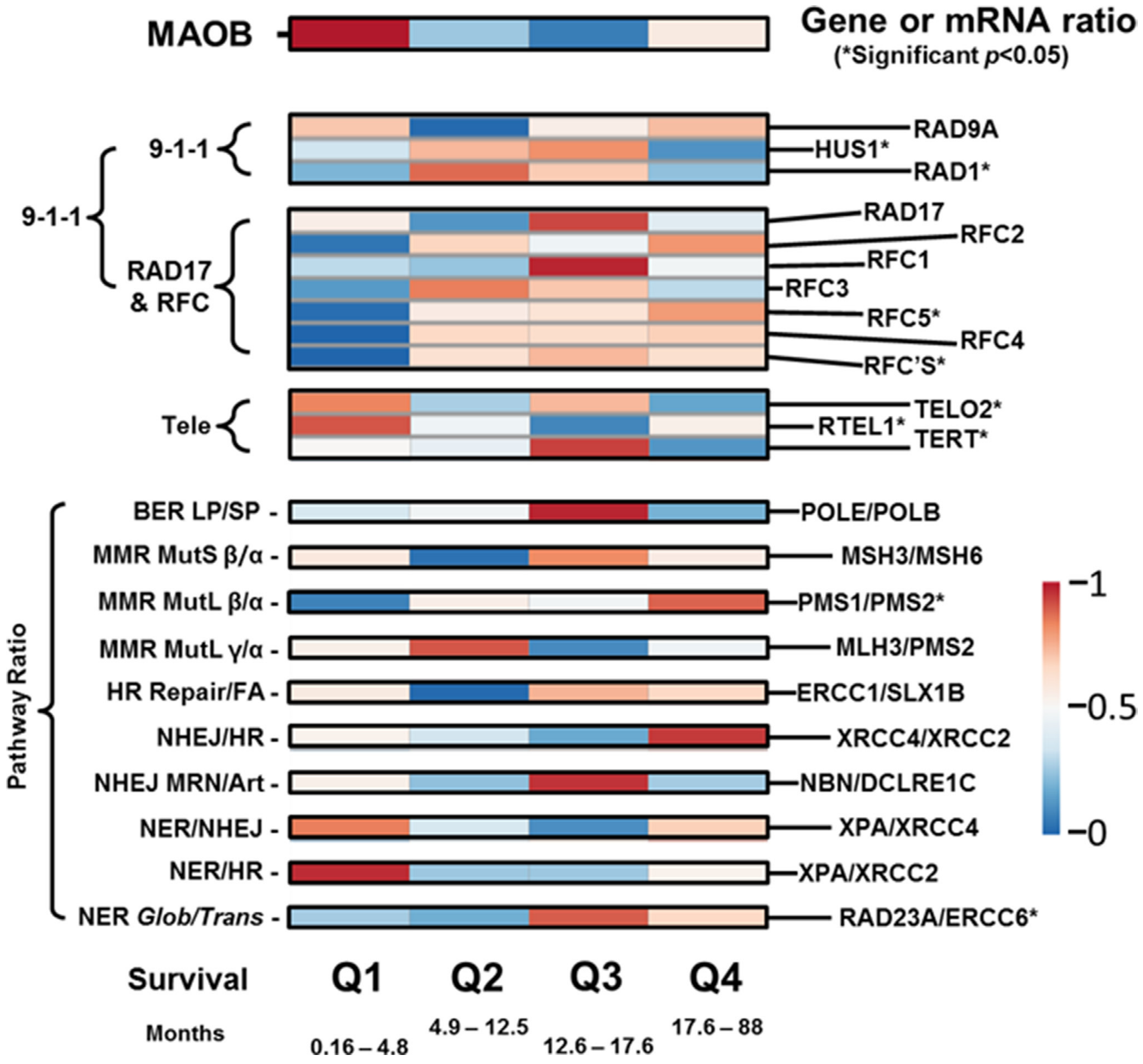

Supplementary Figure 4: DNA repair pathway ratios as a function of GBM patient survival. Supplementary Figure 4 shows a heat map of mRNA levels of DNA repair enzymes, striated by GBM patient outcome. At the top of the heat map is shown the relative levels of MAOB. Shown are the main enzymes that drive the 9-1-1 pathway and the telomere extension pathway enzymes. The ratios of mRNA of key enzymes of different pathways were calculated and are presented. These ratios indicate which DNA repair pathways are up-/down- regulated in different survival cohorts.

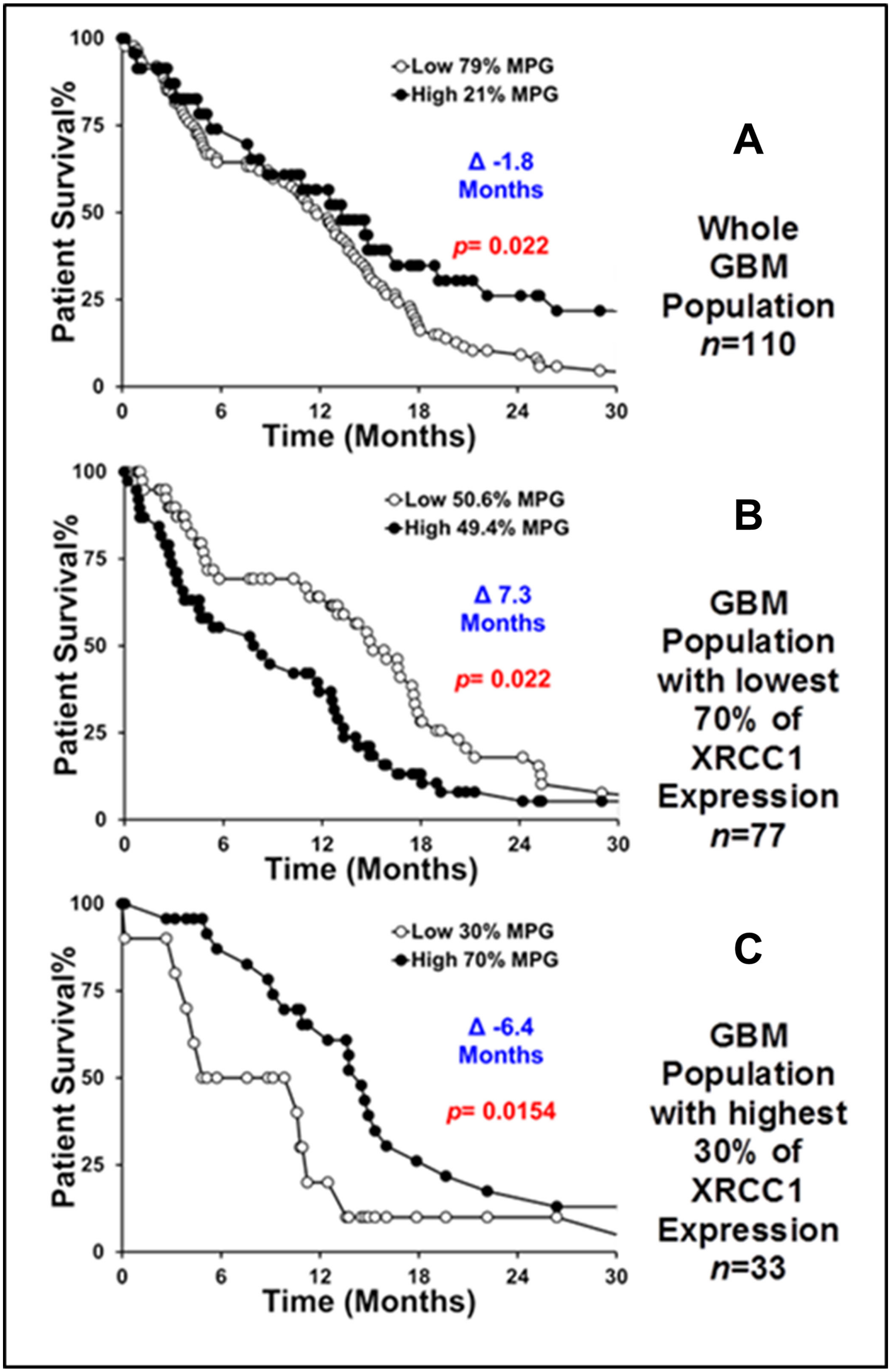

Supplementary Figure 5: Patient survival with respect to MPG expression and stratified by XRCC1 (SP-BER) expression. Supplementary Figure 5 presents the survival curves of three groups of patients, with respect to MPG levels. (**A**) indicates that in the whole $n = 110$ patient population we find that the 79% of the patients with low levels of MPG have a poorer outcome than those with higher levels of MPG. The population was split into two subpopulations with either low or high XRCC1, linked to low and high BER-SP activity. In the $n = 77$ subpopulation of patients where we expect long patch BER to be favored over short patch BER, we find that HIGH MPG is a big risk factor and that the median survival between high and low MPG in this subpopulation is 7.3 months, (**B**). In the smaller population, $n = 33$, where short patch BER is likely to be highly active MGP we find that LOW MPG is a risk factor and that the median survival between the two groups is 6.4 months, (**C**).

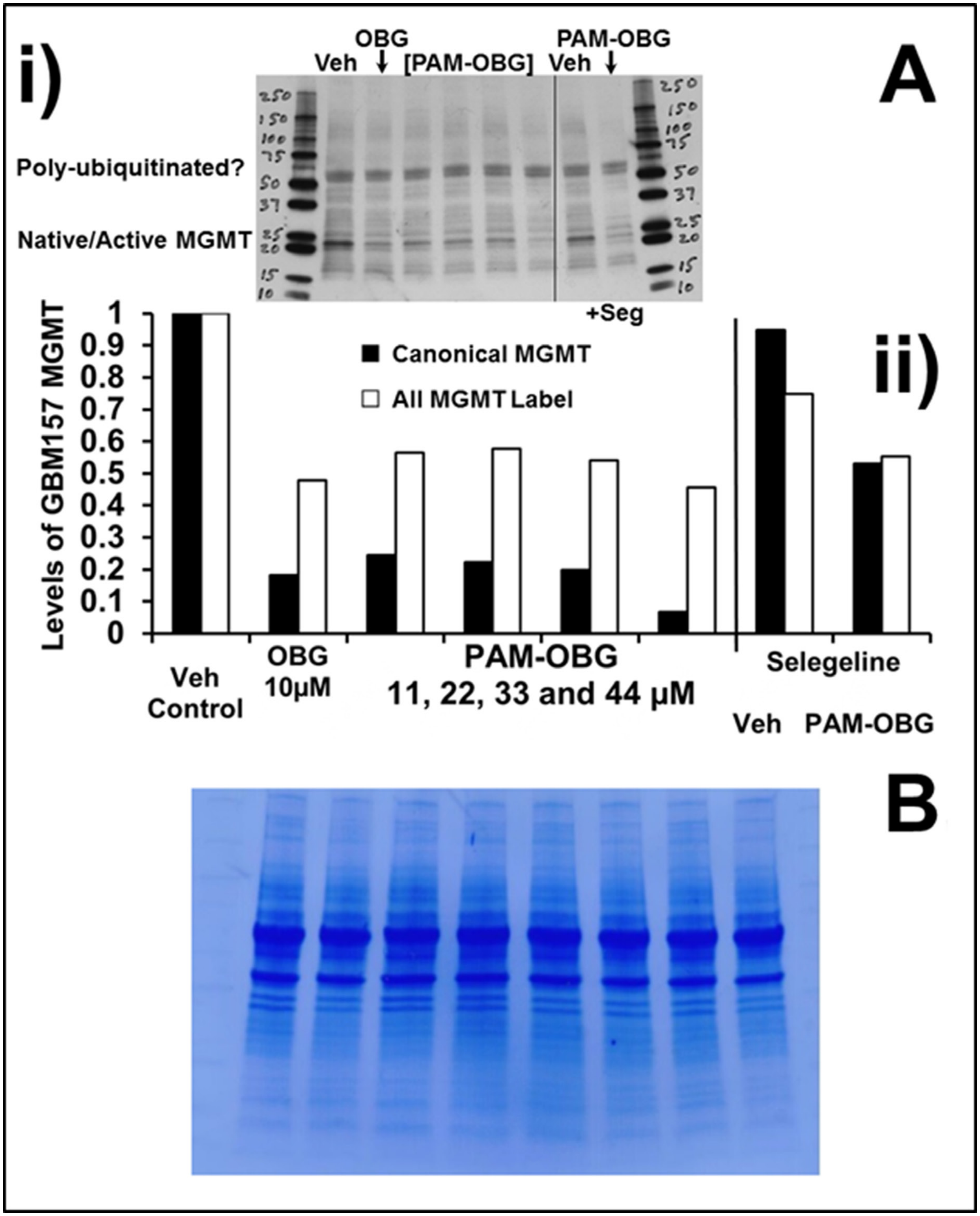

Supplementary Figure 6: Changes in MGMT following incubation with PAM-OBG using Western blot labeling. In Supplementary Figure 6, we present some initial data we generated by incubating GBM157 cells, grown in T-75 tanks, with PAM-OBG, O^6BG, and selegiline and then probing for MGMT using Western blots. (**A**) shows the labeling of MGMT on a single gel, of GBM157 cells incubated in T-75 flasks with DMSO (vehicle), 10 µM O^6BG, 11–44 µM PAM-OBG and 125 µl PAM-OBG, 10 µM selegiline ± 44 µM PAM-OBG. Cells were harvested at 24 hours incubation and run on denaturing a gel, transferred, probed with MGMT IgG and visualized. The bar chart (**B**) indicates that 24 hours of incubation with 33–44 µM PAM-OBG gives similar drops in native, low molecular weight, MGMT in these cells equal to 10 µM O^6BG. Incubation with the MAOB inhibitor causes a slight drop in total MGMT levels and does not completely arrest the loss of active MGMT in the presence of 44 µM O^6BG. (B) shows the Coomassie Blue staining of the developed nitrocellulose and shows no differences in the protein levels in any of the lanes.

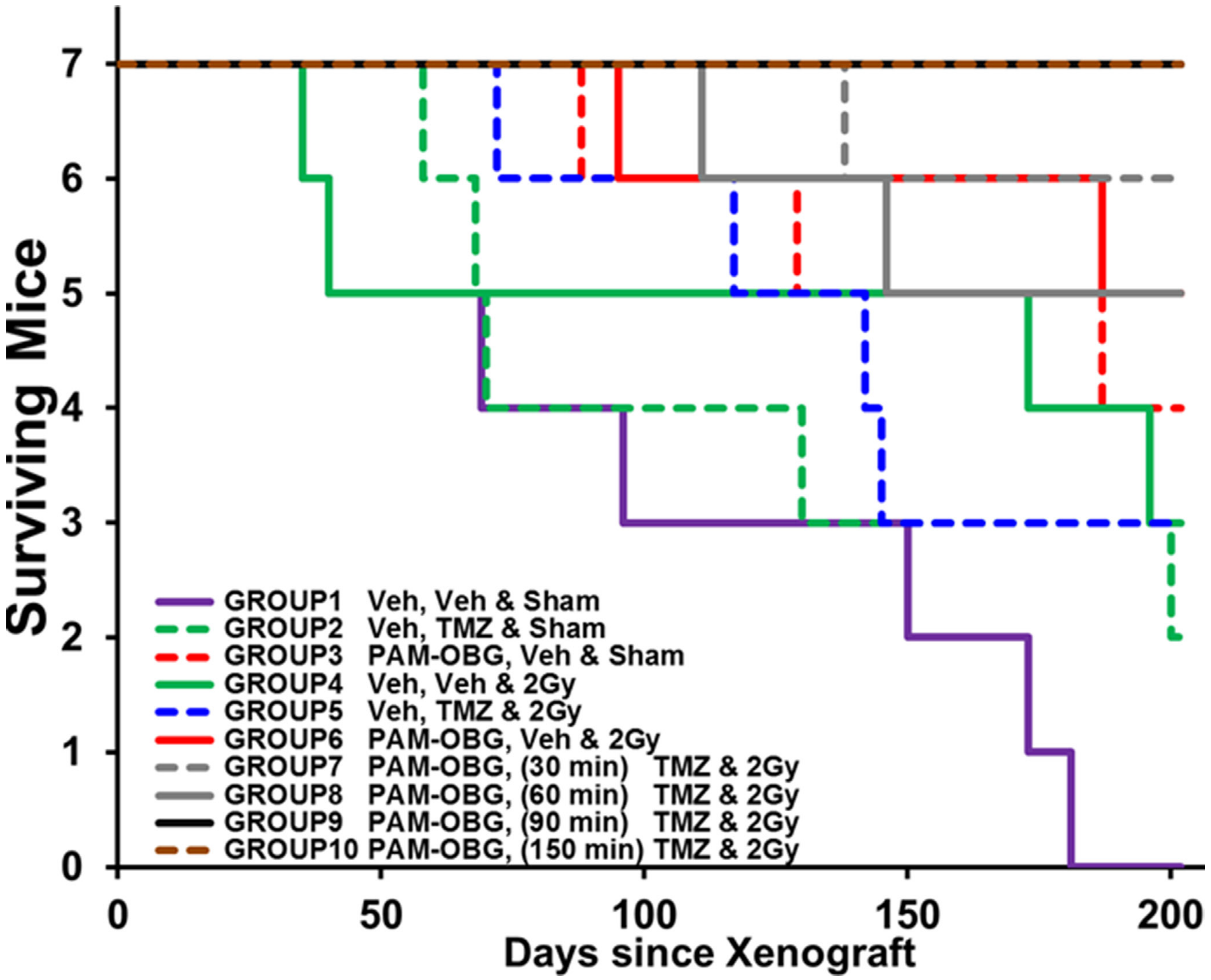

Supplementary Figure 7: Resolving the optimum time between delivery of PAM-OBG and then TMZ/radiotherapy in intracranial models. In Supplementary Figure 7, we present an intracranial glioma mouse model study where we sought to determine the best separation interval between giving PAM-OBG before TMZ, prior to radiotherapy. The mice were given xenografts in the same manner as in the studies presented in Figure 5, except that the inoculum used flank tumor diluted 1/16, rather than 1/4. The six control groups and four treatment groups were given a single treatment, day 8 after xenografts. The dosages were 50 mg/Kg TMZ, 5 mg/Kg PAM-OBG and 2Gy of radiation from a cesium source. We gave the PAM-OBG some 30, 60, 90 and 150 min before giving TMZ by gavage. The survival curves clearly show that the animals that were given PAM-OBG/TMZ/2Gy with the two drugs given between 90–150 mins all survived to the end of the trial. From this study we learned that we should use a 120-minute difference between giving PAM-OBG and TMZ, and that using this very high TMZ dose (in Fox nudes), chemoradiotherapy performed worse than did radiotherapy.

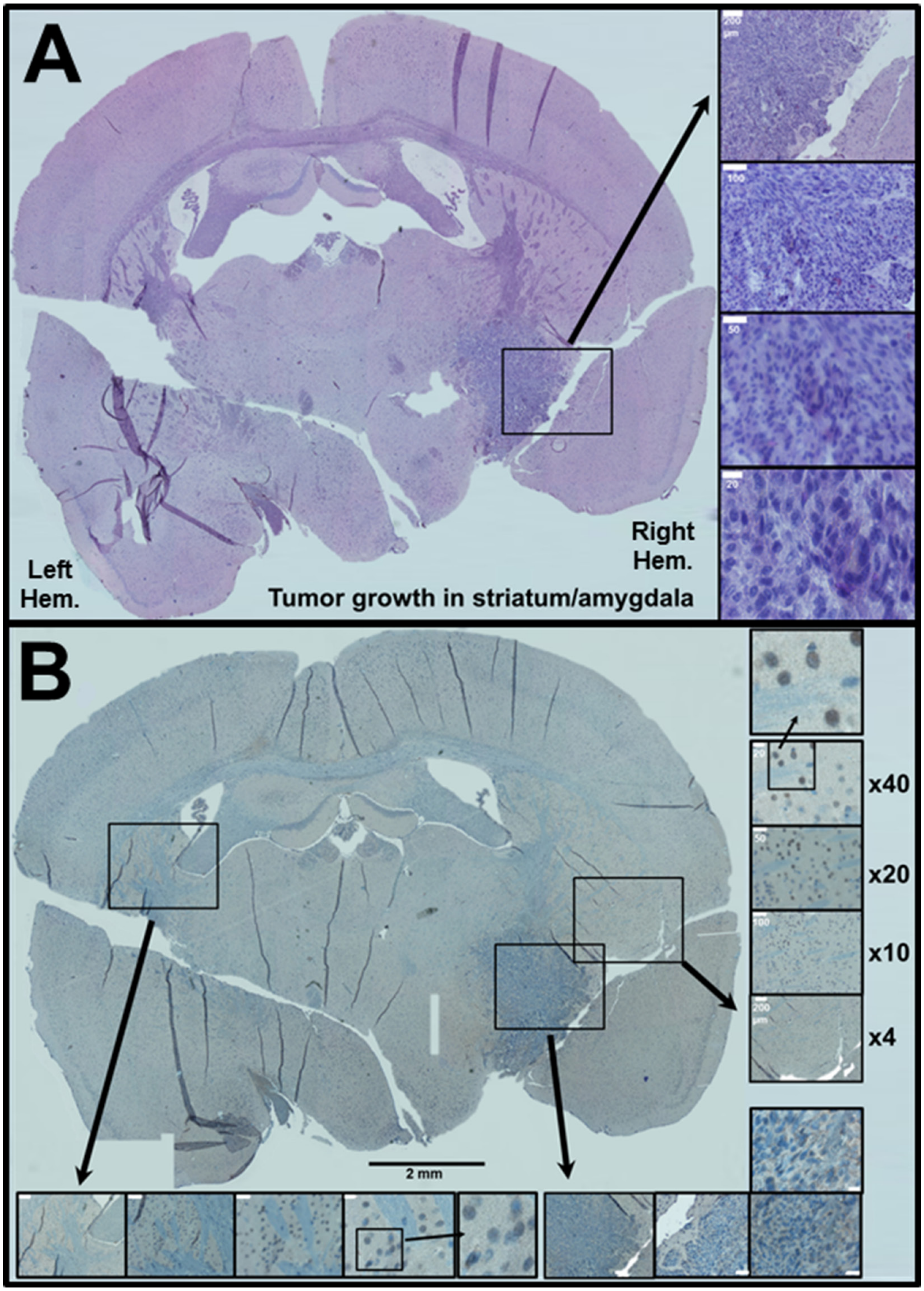

Supplementary Figure 8: Histology of typical GBM157 in control mouse brain. The brain of the first control from the study in Figure 5B was removed and placed in paraformaldehyde immediately after the animal was euthanized upon reaching an ethical endpoint. The brain was dehydrated, waxed, sliced, mounted, rehydrated and underwent epitope retrieval and background peroxidase ablation as previously described [57]. The images shown in (**A** and **B**) are reconstructed montages generates from > 20 images taken at a magnification of ×4. As a section of the left-hand side of the brain was somewhat displaced, we moved its position electronically in both images. In (A) we show a mid-brain slice that has been stained with both hematoxylin and eosin (H&E). A large tumor mass can be seen in the right hemisphere, in the striatum and penetrating into the amygdala. The inserts show images taken at ×4, ×10, ×20 and ×40 magnification, and the scale bars are calibrated at 200, 100, 50 and 20 µm, respectively. (B) shows a slice that was labeled with (anti-human) V9 anti-vimentin mouse monoclonal antibody and visualized using DAB. Nuclei were counter-stained with hematoxylin. There are three boxed regions where we examined an area at increasing magnification. It is clear that the tumor is labeled for V9, and we can see individual labeled glioma cells near the tumor mass. However, the box over the area near the ventricle on the uninjected hemisphere also shows individual DAB positive, human, cells and this shows how penetrating this primary glioma cell line is.
